# Metagenomics comparison identifies shared pathogenic microbiome in humans, pigs and chickens

**DOI:** 10.1007/s00253-026-13948-1

**Published:** 2026-07-08

**Authors:** Sung J. Yu, Dragana Stanley, Thi T. H. Van, Jason C. Steel, Yadav S. Bajagai

**Affiliations:** 1 Institute for Future Farming Systems, Central Queensland University, Rockhampton, QLD 4702 Australia; 2 School of Health, Medical and Applied Sciences, Central Queensland University, Rockhampton, QLD 4702 Australia; 3 School of Science, RMIT University, Bundoora West Campus, Bundoora, VIC 3083 Australia

**Keywords:** One Health, Metagenomics, Intestinal microbiota, Faecal microbiome, Zoonosis, Zooanthroponosis

## Abstract

**Abstract:**

Integrating human, animal, and environmental health is crucial for combating infectious diseases, as an estimated 60 to 75% of emerging infectious diseases originate from zoonotic sources globally. In this study, we analysed 1274 shotgun metagenomic faecal samples of humans, pigs, and chickens collected across multiple countries to estimate levels of microbial sharing at the species-level genome bins (SGBs) resolution. We confirm that host species, rather than geography, significantly structures the gut microbial community, as shown by alpha and beta diversity analyses. Despite this high host specificity, we identified substantial cross-host sharing of SGBs, including taxa recognised as pathogens such as *Escherichia coli*, *Clostridium perfringens*, *Clostridium innocuum*, *Clostridium disporicum*, *Enterococcus* species, and* Streptococcus alactolyticus.* Core taxa were predominantly host-specific, while non-core taxa were more frequently shared across humans, pigs, and chickens. LEfSe analysis identified distinctive microbial signatures for each host, further supporting differences in community composition. These findings demonstrate that unrelated and geographically distant humans and livestock can harbour highly similar microbial populations with pathogenic potential. This work provides molecular evidence supporting the need for integrated One Health surveillance to better detect, manage, and prevent zoonotic and reverse zoonotic transmission events across interconnected human, animal, and environmental systems.

**Key points:**

• *There is substantial cross-host sharing of species-level genome bins, including potential pathogens*

• *Core taxa are predominantly host-specific*

• *Non-core taxa are more likely to be shared across humans, pigs, and chickens*

**Supplementary Information:**

The online version contains supplementary material available at https://doi.org/10.1007/s00253-026-13948-1.

## Introduction

Livestock industries are undergoing rapid technological advancement driven by developments in metagenomics, microbiota, improved breeding and genomics, enhanced animal welfare, and increased automation and robotics. Precision feeding and sensor technologies now support optimised nutrition and continuous animal health monitoring. Together, these innovations increase productivity and sustainability and enhance global food security in countries that can afford to keep pace with research and development.

Global meat consumption continues to increase, driven by rising incomes in middle- and lower-income countries, while more developed countries appear to have reached their peak consumption (Henchion et al. 2021). Although livestock production supports global nutrition and contributes to food safety, it is increasingly scrutinised for its environmental impacts. Major concerns include greenhouse gas emissions, water and land use, deforestation, water and soil pollution, increased antibiotic resistance, and zoonotic disease risk (Lee et al. 2025; Takacs et al. 2025).

The One Health paradigm is an integrated approach to balancing and optimising the health of people, animals, and the environment. This holistic approach recognises the interconnectedness of human, animal, and environmental health, where changes within one system influence the others. The importance of One Health is underscored by the global health threat posed by emerging infectious diseases (EIDs), with an estimated 60 to 75% of all EIDs having a zoonotic origin (Borham et al. 2025; Horefti 2023). The spread of antimicrobial resistance illustrates these connections, with resistance factors originating from livestock manure or human waste entering water systems, spreading to humans and animals, and contributing to disease outbreaks and environmental pollution (Abeles 2024; Stanley et al. 2022).

Livestock production is associated with a high pathogen load, faecal contamination, and numerous zoonotic threats. Poultry-associated diseases, including salmonellosis and campylobacteriosis, are commonly transmitted through contaminated food and faecal material (Agunos et al. 2016; Varela et al. 2022). Critical pathogens, such as avian influenza and Newcastle disease, also pose substantial risks to both animal and human health (Agunos et al. 2016; Varela et al. 2022).

Reverse zoonosis, or zooanthroponosis, involves the transfer of pathogens from humans to animals, and is rising due to urbanisation, globalisation, and climate change. A growing body of research documents viral, bacterial, parasitic, fungal, and protozoal transmission from humans to animals (Al Noman et al. 2024). Despite its importance, reverse zoonosis remains less recognised than traditional zoonotic transmission. The potential for human pathogens to infect large numbers of farmed animals raises concerns about animal welfare, economic losses, and the amplification of pathogens that may subsequently return to humans. Kibenge (2023) suggested a One Health approach to addressing these issues, including active surveillance and vaccination of humans, especially those exposed to susceptible livestock.

Recent advances in sequencing technologies enable the culture-free identification of pathogens and can facilitate the reconstruction of microbial genomes from metagenomic data. Such approaches enable the identification of strains transmitted from livestock to farmers and vice versa, even when strains are not overtly pathogenic. Although awareness of zoonotic transmission is common among farmers, understanding of reverse zoonosis remains limited (Al Noman et al. 2024; Kibenge 2023).

Currently, most journals and funding agencies require deposition of raw sequencing data in public repositories, allowing large cross-species metagenomic datasets to be retrieved and reanalysed. This enables comparative investigation of reconstructed species-level genome bins (SGBs) to identify microbial lineages shared among host species (Borderes et al. 2021).

Multiple strain-level genome bins can be assigned to the same species but map to distinct metagenomes and are considered approximations of strain-level. Once a species-level genome bin is identified, strain-level analysis goes deeper into genetic differences, identifying single-nucleotide polymorphisms (SNPs) or the presence/absence of specific genes (e.g. virulence factors or antibiotic resistance genes) within that species SGB, creating so-called sub-bins. Although exact strain-level resolution remains technically challenging, comparing these unique metagenomic SGBs provides insight into the likelihood of shared microorganisms and potential cross-host transmission risks.

In this study, we downloaded publicly available shotgun metagenomic sequencing studies on chicken, pigs, and humans from studies originating from Belgium, Bulgaria, Canada, China, Denmark, France, Germany, India, Italy, Malawi, Netherlands, Poland, South Korea, Spain, the UK, and the USA, ensuring multicontinental coverage. After quality filtering, 1274 faecal metagenomic samples were analysed; pigs and humans were well represented with 537 and 539 samples respectively, whereas only 198 chicken samples were included because faecal samples are less commonly available in poultry research, where cloacal swabs and caecal samples are more frequently used. Faecal samples were selected to enable a consistent cross-host comparison and because faecal material is a major route through which many enteric microorganisms, including zoonotic and opportunistic pathogens, can be shed into farm, food-chain and environmental interfaces (Delahoy et al. 2018; Penakalapati et al. 2017). Our objectives were to quantify the magnitude of shared SGBs among the three hosts, evaluate the occurrence of taxa with known pathogenic potential, and interpret these data within a One Health framework relevant to zoonotic and reverse-zoonotic risk assessment.

## Materials and methods

### Study design and data sources

We conducted a cross-host species comparative analysis of the faecal microbiomes from humans, chickens, and pigs using publicly available shotgun metagenomic datasets. To ensure a like-for-like comparison, only faecal samples were included, resulting in a smaller number of chicken samples. Studies were selected to create balanced cohorts spanning multiple countries and independent research groups. Raw sequencing data and associated metadata were retrieved from public repositories (primarily the NCBI Sequence Read Archive, SRA) using the SRA Toolkit.

A total of 1274 samples were analysed: human (*n* = 539), pig (*n* = 537) and chicken (*n* = 198) (Table 1). Countries represented were China, Italy, and the UK for chicken; Germany, India, Italy, Malawi, the Netherlands, Poland, South Korea, and the USA for human; and Belgium, Bulgaria, Canada, China, Denmark, France, Germany, Italy, the Netherlands, Poland, Spain, and the UK for pig. Datasets were selected based on the quality of sequences and annotations.

**Table 1:** List of studies included in the analysis

NCBI accession ID	Number of samples	Country of origin	Host
PRJEB21076	62	Italy	Chicken
PRJNA586747	60	China	Chicken
SRP083441	29	China	Chicken
PRJNA543206	47	UK	Chicken
PRJEB62041	133	India and Malawi	Human
PRJEB64621	81	Netherlands	Human
PRJNA1001944	152	Poland	Human
PRJNA1004651	10	USA	Human
PRJNA1011235	29	Germany	Human
PRJNA1036590	44	Italy	Human
PRJNA1049470	75	USA	Human
PRJNA1093257	15	South Korea	Human
PRJEB11755	121	Denmark and China	Pig
PRJEB22062	185	Netherlands, Germany, France, Spain, Belgium, Italy, Poland, Denmark, Bulgaria	Pig
PRJEB34736	26	UK	Pig
PRJEB31742	3	China	Pig
PRJNA629856	167	Canada	Pig
PRJNA737271	12	Canada	Pig
PRJNA791695	23	China	Pig

## Read processing and quality control

Initial quality control of paired-end FASTQ files was performed using FastQC v0.11.2 (Andrews 2010) and MultiQC v1.11 (Ewels et al. 2016), followed by adapter removal and quality trimming with fastp using default parameters, including automatic adapter detection and the default quality and length filtering thresholds (Chen et al. 2018). Host-derived reads were removed using KneadData v0.7.10 (https://huttenhower.sph.harvard.edu/kneaddata) employing Bowtie2 v2.5.1 (Langmead and Salzberg 2012) to align reads against species-specific host genomes for human (GRCh38.p14, Accession: PRJNA31257), chicken (bGalGal1, Accession: PRJNA660758), and pig (Sscrofa11.1, Accession: PRJNA13421) to retain predominantly microbial reads for downstream profiling. After processing, approximately 5.9 × 10^1^ high-quality, host-filtered reads were retained across all samples, with an average of ≈ 46.30 million reads per sample. Sample-level accession numbers with the number of quality-filtered reads for each sample are presented in Supplementary Table S1.

## Bioinformatics and statistical analyses

The KneadData trimmed clean sequences were analysed for microbial taxonomic profile using MetaPhlAn 3 (Beghini et al. 2021) with default parameters and mpa_vJan21 ChocoPhlAn database. The downstream statistical analysis and visualisation were done with the R program v4.4.2 using the packages Phyloseq v1.50.0 (McMurdie and Holmes 2013) and Microeco v1.15.0 (Liu et al. 2021) and Microbiome v1.28.0 (Lahti and Shetty 2018).

Within each host group and at the selected taxonomic rank, taxa were classified as core if they reached ≥ 0.1% relative abundance in ≥ 50% of samples. Taxa that did not meet this prevalence at the same detection threshold were classified as non-core. The same thresholds were applied across the host groups to enable like-for-like comparisons and subsequent overlap analyses. Host-specific lists of taxa classified as core and non-core, and the overlap among humans, chickens, and pigs, were quantified by computing pairwise and three-way intersections. Shared taxa were summarised numerically and visualised with Venn diagrams.

## Results

**Fig. 1:**
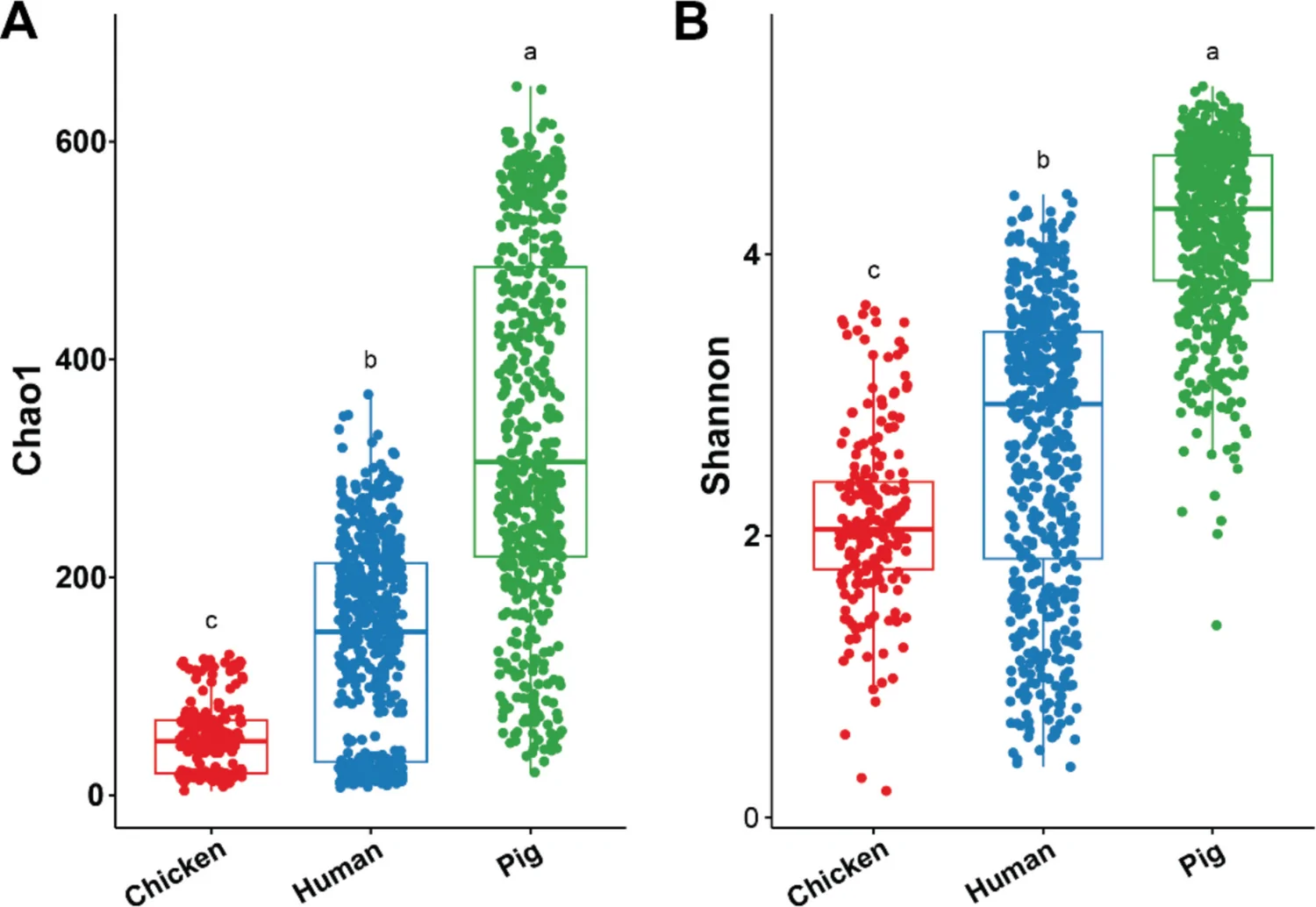
Comparison of microbial alpha diversity indices among chicken (red), human (blue), and pig (green) gut microbiota. **A** Chao1 richness index representing the estimated number of observed species (richness) among hosts, with pigs showing the highest richness, followed by humans and chickens. **B** Shannon diversity index across host species, showing a similar trend. Host species with different lowercase letters (a–c) above the boxplots indicate statistically significant differences between groups (Kruskal–Wallis test followed by Dunn’s post hoc test, *p* < 0.05, applied in the Microeco R package). Alpha diversity was calculated at the species-level genome bins (SGBs) level.

Recent advances in our understanding of the roles of intestinal microbiota suggest that differences in gut anatomy and environmental factors influence alpha and beta diversity of intestinal microbiomes. Although the samples used in this study originated from different countries and studies, consistent patterns emerged when comparing the three host groups. Alpha diversity analysis showed that richness, measured using the Chao1 index, and diversity, measured using the Shannon index, varied among chickens, humans, and pigs (Fig. 1). Chicken samples in our study had the lowest richness and diversity, significantly lower than those of humans and pigs. In contrast, pig samples showed the highest levels of both richness and diversity, but also the greatest dispersion among samples, indicating substantial heterogeneity across pig datasets. The lower alpha diversity observed in chickens may reflect biological differences among host species, but may also have been influenced by the smaller number of available chicken faecal datasets.

Supplementary Figs S1 and S2 show the overall taxonomic composition of the faecal microbiota of chickens, humans, and pigs at the phylum and genus levels, respectively. *Firmicutes* was the dominant phylum in all three host species, but the abundance of other major phyla differed between hosts. Chicken microbiota had higher levels of *Proteobacteria* and higher abundance of genera such as *Escherichia* and *Lactobacillus*. Human microbiota had a higher representation of *Bacteroidetes* and *Actinobacteria*, with greater abundance of genera involved in complex carbohydrate fermentation and short-chain fatty acid production, including *Bacteroides*, *Phocaeicola*, *Alistipes*, *Faecalibacterium*, and *Bifidobacterium*. In contrast, the pig microbiota were dominated by *Firmicutes* and *Bacteroidetes* and showed higher abundances of *Prevotella*, *Lactobacillus*, and *Streptococcus*. Overall, although the major phyla were broadly similar across hosts, clear differences at both phylum and genus levels support strong host-associated structuring of the gut microbiota across the geographically diverse datasets analysed in this study.

**Fig. 2:**
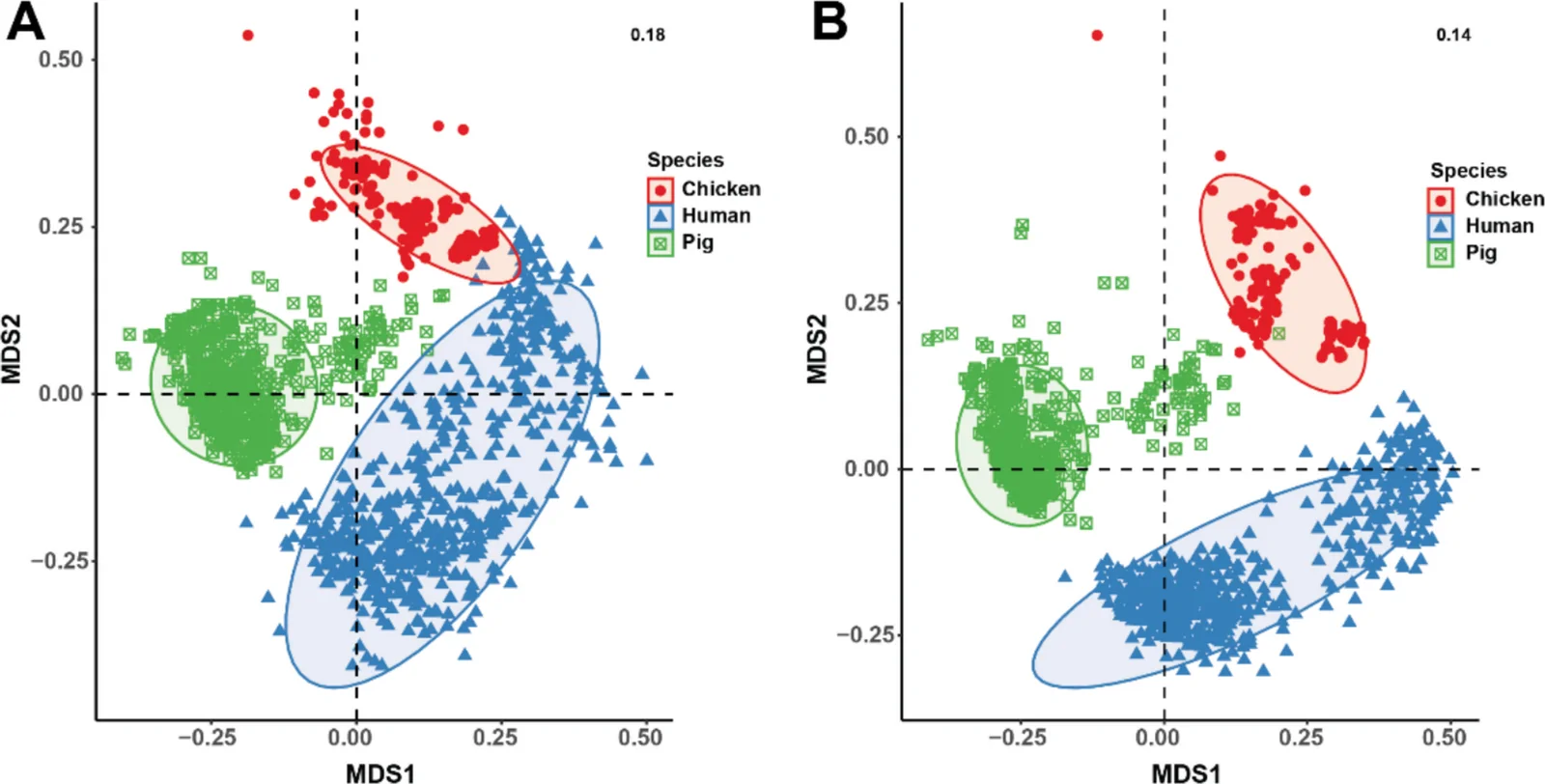
Beta diversity of gut microbial communities among chickens, humans, and pigs. Panel **A** shows a non-metric multidimensional scaling (NMDS) ordination based on the Bray–Curtis distance matrix, which represents compositional dissimilarity in relative abundance among samples. Panel **B** presents NMDS results derived from the Jaccard distance matrix, reflecting dissimilarity in community membership based on presence-absence (qualitative) data. Each point corresponds to an individual sample, coloured by host species, and closer points indicate more similar microbial community structures.

Bray–Curtis dissimilarity and Jaccard distance were used to assess beta diversity. Bray–Curtis considers differences in taxon abundance and is more influenced by dominant taxa, whereas Jaccard distance is based on presence-absence patterns and is more sensitive to rare taxa. Both measures showed a similar pattern, with SGBs-level microbial communities clustering mainly by host species rather than by country of origin or geographic region (Supplementary Fig. S3 and Fig. 2).

Despite the considerable geographic distance and study methodological differences, the Fig. 2 plot reveals clear clustering patterns by host type, demonstrating that both microbial composition (Bray–Curtis) and membership (Jaccard) differ significantly (PREMANOVA *p* < 0.001) among chickens, humans, and pigs, indicating that host species was a major driver of faecal microbiota structure in this dataset.

**Fig. 3:**
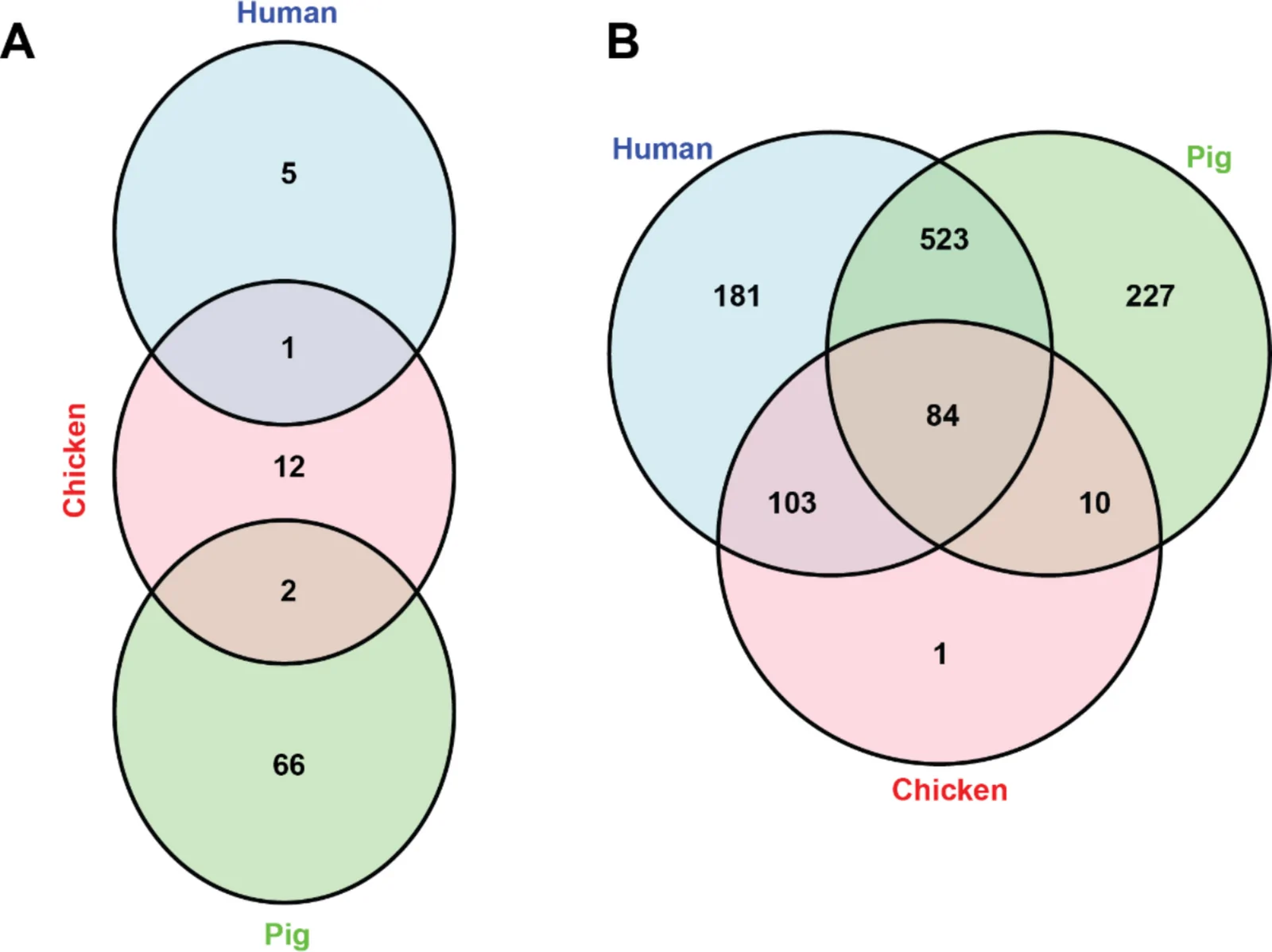
Core and non-core species-level genome bins (SGBs) among the gut microbiota of chickens, humans, and pigs. Panel **A** shows the core taxa, defined as taxa with ≥ 0.1% relative abundance in ≥ 50% of samples within each host group. Panel **B** illustrates non-core taxa, comprising taxa that did not meet this abundance and prevalence threshold

Core and non-core species are the most and least abundant in the community, respectively, and their membership depends heavily on the analysis cutoffs described in the methods. Figure 3 shows the core and non-core species-level genome bins (SGBs).

**Fig. 4:**
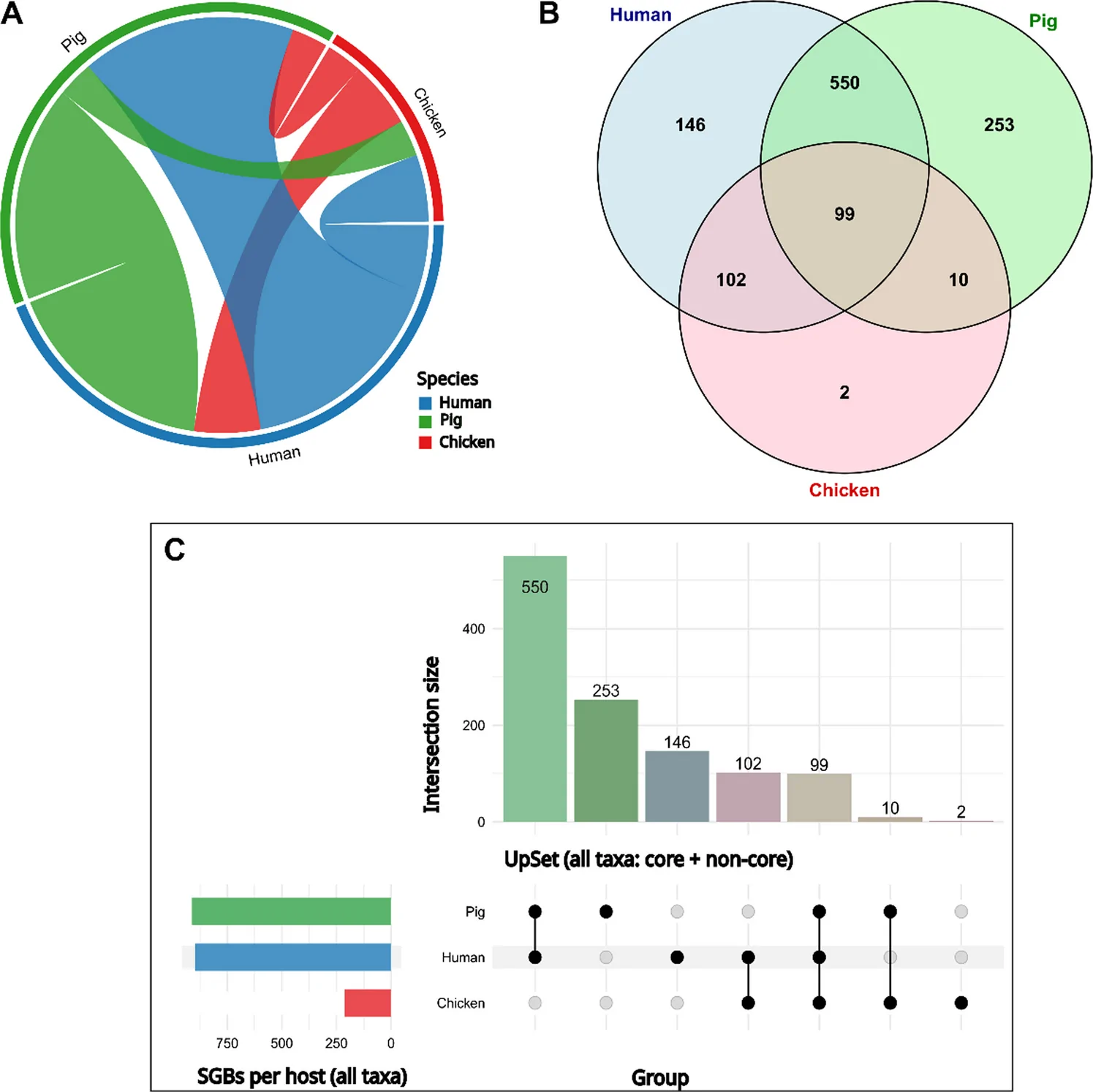
Shared SGBs among the faecal microbiota of chickens, humans, and pigs. **A** Chord diagram showing shared SGBs among host species. The outer coloured arcs represent the total SGB pool detected in each host, and the connecting ribbons represent SGBs shared between hosts. Ribbon width is proportional to the number of shared SGBs. **B** Venn diagram showing the number of SGBs unique to each host and shared between host combinations. **C** UpSet plot showing the same host-specific and shared SGB intersections as in panel **B**. The vertical bars indicate intersection size, the connected black dots below each bar indicate the host combination represented, and the horizontal bars show the total number of SGBs detected per host. Vertical bar colours correspond to the matching intersections shown in the Venn diagram in panel **B**, while the horizontal bar colours correspond to host species colours used in panel **A** (blue, human; green, pig; red, chicken).

Figures 3 and 4 show that core SGBs are highly host-associated, whereas non-core species are more commonly shared among multiple host species, noting that each host species has a different total number of SGBs, using the current dataset.

**Fig. 5:**
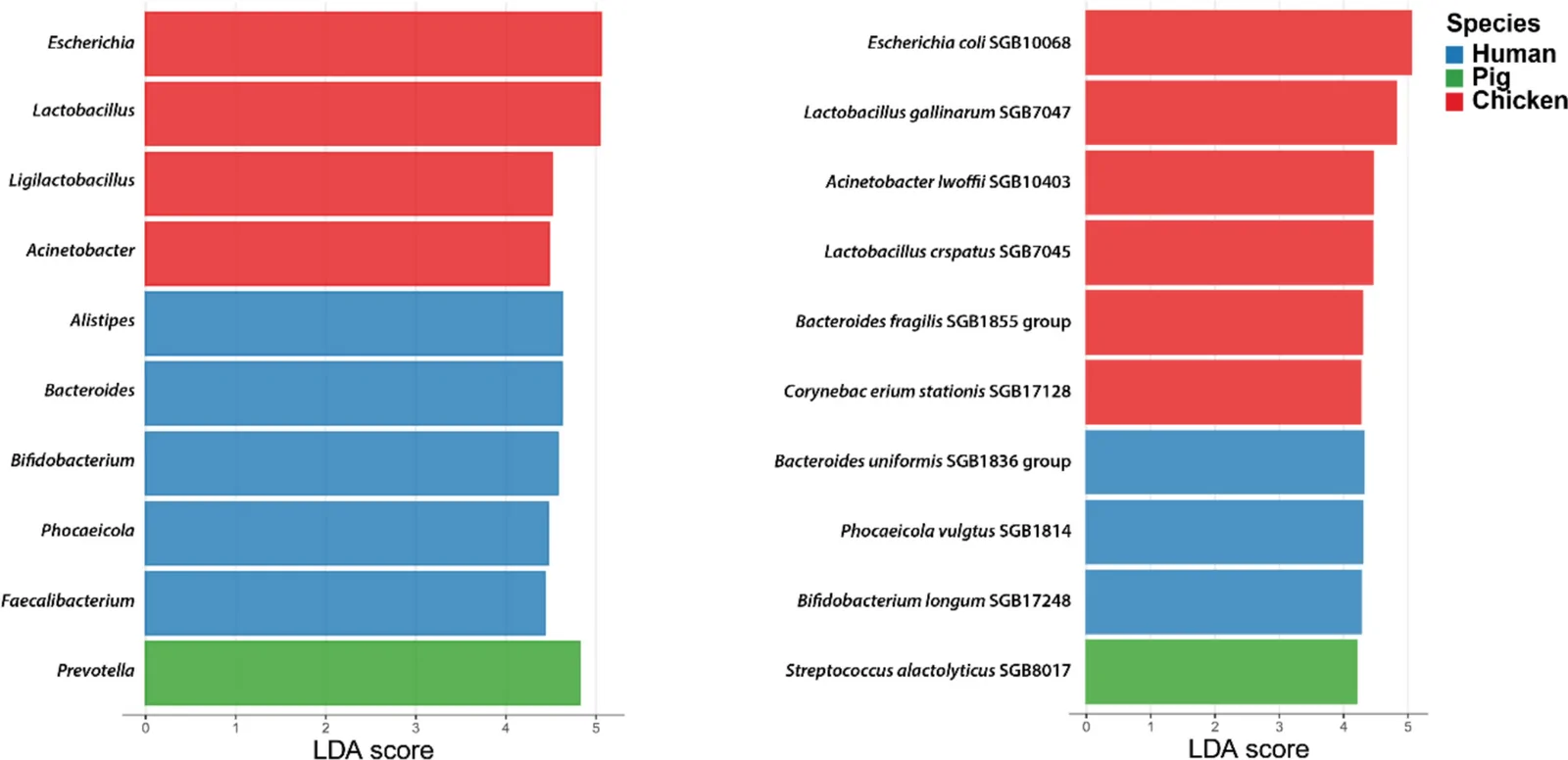
LEfSe analysis showing genus and species (SGBs)-level biomarker microbial taxa that characterise pig, chicken, and human faecal microbiota.

Linear discriminant analysis effect size (LEfSe) analysis (Fig. 5) was used to identify microbial biomarkers that differ between the host species. We acknowledge that, due to fewer chicken samples, LEfSe would have lower discriminative ability for identifying chicken-relevant taxa compared to human- and pig-relevant biomarkers; however, the relatively high number of chicken samples (*n* = 198) and their diversity and robustness of the LEfSe algorithm should also be noted.

Chickens had characteristic high levels of *Proteobacteria* and *Fusobacteria* phyla, as well as the genera *Escherichia*, *Lactobacillus*, *Acinetobacter*, and *Ligilactobacillus* (Fig. 5). Human characteristic phyla were *Actinobacteria* and *Verrucomicrobia*, with *Alistipes*, *Bacteroides*, *Bifidobacterium*,* Phocaeicola*, and *Faecalibacterium* as distinct genera, while in pigs, characteristic phyla include *Spirochaetes* and *Chlamydiae*, with *Prevotella* genus as the only identified characteristic pig genus.

## Discussion

This study provides a multicontinental comparison of faecal metagenomic datasets from humans, pigs, and chickens. It shows that host species strongly shapes gut microbial communities, even when samples come from different countries and independent studies. This finding is consistent with previous studies showing that host physiology, diet, intestinal anatomy, and immune selection influence gut microbiome composition (Degregori et al. 2025; Wilde et al. 2024). However, the detection of shared species-level genome bins across species suggests that host-associated structuring does not fully prevent some microbial lineages from occurring across host boundaries.

The difference between core and non-core taxa was important in this study. Core species-level genome bins were mainly linked with specific host species. This suggests that common and dominant faecal microorganisms are strongly influenced by host ecology. In contrast, non-core taxa were more often shared among humans, pigs, and chickens. This pattern suggests that taxa with low or moderate prevalence may play a larger role in microbial overlap between hosts. These taxa may include transient microorganisms, environmentally acquired microorganisms, food-chain-associated taxa, or opportunistic lineages that can colonise more than one host species.

The livestock industry encompasses multiple animal species, including poultry, pigs, and ruminants, and provides meat, eggs, milk, and other products essential for human health and the global economy. Despite these contributions, the industry faces continually evolving challenges that demand improvements in productivity, sustainability, and biosecurity. Biosecurity has always been at the forefront of livestock management, and new threats continue to arise through rapid globalisation, increased movement of people and goods, and the introduction of exotic diseases. The industry addresses these challenges by maintaining strict hygiene for visitors and equipment, conducting ongoing monitoring of animal health, managing feral animals, especially in free-range settings, and ensuring that response plans are in place for any suspected disease outbreaks.

Human, ecological, and pathogen-related factors all contribute to the rise of emerging and reemerging diseases, with climate change increasingly recognised as an accelerator of these events. Emerging infectious diseases are not limited to humans or human-associated animals. Evidence now demonstrates that novel pathogens can arise in a wide range of wild species, including snakes (Liu et al. 2023), birds (Robinson et al. 2010), and insects (Damian et al. 2020). These observations highlight the need for integrated approaches that recognise the interconnectedness of environmental and biological systems.

Livestock, including pigs, poultry, and cattle (Astill et al. 2018; Grout et al. 2022; McLean and Graham 2022), have been identified as reservoirs for the amplification of emerging pathogens that can pose a risk to humans. Traditional assumptions suggesting that most microbial species evolved toward a single preferred host are being challenged by modern genomic technologies, which reveal that many pathogens can infect multiple hosts or undergo host switching. Host switching is a significant factor in the emergence of new infectious diseases. Chavez-Dozal et al. (2014) examined gene exchange between marine pathogen strains isolated from different host species on different continents. They showed that gene complementation between strains increased the fitness of *Vibrio fischeri*, enabling it to achieve competitive dominance in a non-native host. Similarly, humans have been identified as a central reservoir for the multi-host bacterial pathogen, *Staphylococcus aureus*, in which gene exchange between ancient and contemporary strains has facilitated host-switching events that contributed to the emergence of livestock-adapted lineages (Richardson et al. 2018). These examples highlight the potential for both zoonoses and zooanthroponosis to occur within farm environments.

Other factors that increase the ability of pathogens to jump from one host to another include the presence of multi-host pathogens, the presence of mutagens in feed and the environment, and the innate adaptability of microbial pathogens (Richardson et al. 2018). Environmental mutagens are not decreasing, and climate change may increase mutation rates by elevating temperatures and influencing molecular processes related to DNA stability (Jacobs 2021; Liao et al. 2024). These conditions heighten the risk that human or livestock outbreaks may provide opportunities for pathogen adaptation and cross-species transmission.

Because this analysis cannot distinguish pathogenic from benign SGBs, this study focused specifically on shared SGBs associated with known or emerging pathogens (Table 2, and Supplementary Table S2). Several shared SGBs were identified across humans, pigs, and chickens, including *Escherichia coli* SGB10068, *Clostridium perfringens* SGB6191, *Clostridium disporicum* SGB6172, *Clostridium innocuum* SGB4037 group, *Enterococcus cecorum* SGB7961,* Enterococcus gallinarum* SGB7951, and *Streptococcus alactolyticus* SGB8017*.* Each of these species includes strains capable of causing disease in humans or animals, or both, but also includes non-pathogenic or commensal strains.

**Table 2:** Shared species-level genome bins (SGBs). The table excludes unclassified genera and hypothetical genera. Please note that some species have multiple SGBs.

Shared (H + P + C)
*Alistipes ihumii* SGB2328
*Alistipes putredinis* SGB2318
*Anaerofustis stercorihominis* SGB49059
*Anaerotignum lactatifermentans* SGB5193
*Anaerotruncus colihominis* SGB14963
*Bacteroides caccae* SGB1877
*Bacteroides fragilis* SGB1853
*Bacteroides fragilis* SGB1855 group
*Bacteroides ovatus* SGB1871
*Bacteroides thetaiotaomicron* SGB1861
Bacteroides togonis SGB1822
*Bacteroides uniformis* SGB1836 group
*Bacteroides xylanisolvens* SGB1867
*Bifidobacterium adolescentis* SGB17244
*Bifidobacterium pseudolongum* SGB17279
*Butyricicoccus porcorum* SGB14982
*Candidatus Avimicrobium caecorum* SGB15350
*Citrobacter freundii* SGB10083 group
*Clostridium disporicum* SGB6172
*Clostridium disporicum* SGB6178
*Clostridium innocuum* SGB4037 group
*Clostridium perfringens* SGB6191
*Corynebacterium xerosis* SGB17082
*Desulfovibrio piger* SGB15467 group
*Dysosmobacter welbionis* SGB15078
*Eggerthella lenta* SGB14809
*Enorma massiliensis* SGB14749
*Enterobacter hormaechei* SGB10130 group
*Enterococcus cecorum* SGB7961
*Enterococcus faecalis* SGB7962
*Enterococcus faecium* SGB7967
*Enterococcus gallinarum* SGB7951
*Erysipelatoclostridium ramosum* SGB6744
*Escherichia coli* SGB10068
*Eubacterium rectale* SGB4933 group
*Evtepia gabavorous* SGB15120
*Faecalibacterium prausnitzii* SGB15316 group
*Faecalibacterium prausnitzii* SGB15342
*Faecalicoccus pleomorphus* SGB6791 group
*Flavonifractor plautii* SGB15132
*Hydrogeniiclostidium mannosilyticum* SGB14890
*Intestinimonas timonensis* SGB29334
*Klebsiella pneumoniae* SGB10115
*Lactobacillus amylovorus* SGB7050
*Lactobacillus crispatus* SGB7045
*Lactobacillus delbrueckii* SGB7020
*Lactobacillus johnsonii* SGB7041
*Lactobacillus vaginalis* SGB7098
*Lactococcus lactis* SGB7985
*Ligilactobacillus salivarius* SGB7082
*Limosilactobacillus fermentum* SGB7106
*Limosilactobacillus oris* SGB7093
*Limosilactobacillus reuteri* SGB7095
*Negativibacillus massiliensis* SGB14912
*Parabacteroides distasonis* SGB1934
*Phascolarctobacterium succinatutens* SGB5765 group
*Phocaeicola coprophilus* SGB1888
*Phocaeicola dorei* SGB1815
*Phocaeicola plebeius* SGB1903 group
*Phocaeicola vulgatus* SGB1814
*Prevotella copri* clade A SGB1626
*Prevotella copri* clade B SGB1613
*Proteus mirabilis* SGB9992
*Pseudoflavonifractor capillosus* SGB15140
*Romboutsia timonensis* SGB6148
*Roseburia faecis* SGB4925
*Ruminococcus bicirculans* SGB4262
*Streptococcus alactolyticus* SGB8017
*Turicibacter sanguinis* SGB6846
*Turicibacter sanguinis* SGB6847
*Victivallis vadensis* SGB9202

The sharing of species-level genome bins for *E. coli*, *C. perfringens*,* Clostridium difficile*, *C. innocuum*, *E. cecorum*, *E. gallinarum*, and *S. alactolyticus* between humans and livestock raises substantial animal and public health concerns that require further pathogenic strain-resolved investigation*. E. coli* is well known for causing urinary tract infections (UTIs) in humans, and represents a considerable health burden that requires prolonged and repetitive use of antibiotics. Broiler meat, broiler chickens, pork, and pigs have been identified as probable sources of UTI strains in humans, with evidence implicating chickens as a significant source of the pathogen (Jakobsen et al. 2010a, 2010b; Schaeffer 2013).

The toxin-producing *C. perfringens* infects humans, pigs, and chickens (Camargo et al. 2024). Various strains can cause significant necrotic enteritis or similar disease in multiple hosts, including humans, pigs, and chickens (Lu et al. 2021). However, the versatility of these strains extends beyond typical enteric diseases to frequently cause fatal neurotoxic conditions in ruminants (Finnie and Uzal 2022). The potential adaptation of certain strains to human hosts represents a growing concern and remains a topic of active scientific debate (Dorca-Arevalo et al. 2008; Finnie and Uzal 2022). Of particular concern is the epsilon toxin (ETX)-producing *C. perfringens*, which is classified as a bioterrorism agent due to its potency. Adaptation of this strain to human hosts could cause enterotoxemia similar to that observed in animals, with devastating implications for public health. Notably, *C. difficile*, a well-characterised and devastating pathogen (Wang et al. 2025), was not identified as a shared SGB in this study, which may reflect its lower prevalence or abundance in faecal metagenomes from the datasets analysed, rather than a true absence from these host species. Additional concerns arise from the emerging pathogens, *C. disporicum* and *C. innocuum. C. disporicum* was recently implicated in the first reported cases of joint and abdominal infections (McBride et al. 2017; Plassart et al. 2013), while *C. innocuum* has only recently been recognised as an emerging pathogen (Cherny et al. 2021; Wu et al. 2023).

Interestingly, our study identified no shared SGBs for several well-known pathogen groups, including *S. aureus*, *Streptococcus suis*, nor SGBs from genera often associated with respiratory infections such as *Haemophilus*, *Moraxella*, *Mycobacterium*, *Bordetella*, *Pseudomonas*, *Neisseria*, *Chlamydia*, and *Legionella.* The absence of shared SGBs across these groups may reflect host specificity but could also be due to insufficient representation in the included datasets.

The LEfSe analysis indicated that chickens had a higher representation of *Escherichia* species, suggesting a higher abundance of these taxa in faecal material. However, it is important to acknowledge that microbiome profiles differ across intestinal regions in each host, and faecal abundance does not necessarily reflect overall intestinal colonisation.

The broader implication of these findings is that One Health surveillance should not be limited to known outbreak pathogens. Shared non-core taxa may give early signals of microbial exchange between humans, livestock, and environments, especially when combined with virulence-gene, antimicrobial-resistance, and strain-level analyses. To translate the One Health framework into action, greater emphasis is needed on implementation science. Implementation science examines the gap between controlled research outcomes and real-world application, which can be influenced by socio-economic and political contexts (Ogunseitan 2022). A lack of multisectoral coordination between veterinary and medical sectors remains a significant challenge (Shafique et al. 2024). Examples from integrated approaches, such as those used in Tick-Borne Disease surveillance in the Netherlands, demonstrate that high transdisciplinary collaboration and public engagement can lead to measurable improvements in health outcomes and early detection of emerging pathogens (Garcia-Vozmediano et al. 2022).

In conclusion, our findings provide clear molecular evidence that humans, pigs, and chickens share multiple microbial taxa, including species with pathogenic potential. These results reinforce the importance of integrated surveillance systems that monitor human, animal, and environmental health simultaneously. Capacity building at local and regional levels, combined with cross-sector collaboration, is essential to support early detection, prevention, and containment of emerging health threats within the One Health framework.

## Supplementary Information

Below is the link to the electronic supplementary material.

ESM 1 (PDF 1.18 MB)

## Data Availability

The data presented in this study are available in the National Library of Medicine’s National Centre for Biotechnology Information (NCBI) at https://www.ncbi.nlm.nih.gov/, under reference numbers: PRJEB21076, PRJNA586747. SRP083441, PRJNA543206, PRJEB62041, PRJEB64621, PRJNA1001944, PRJNA1004651, PRJNA1011235, PRJNA1036590, PRJNA1049470, PRJNA1093257, PRJEB11755, PRJEB22062, PRJEB34736, PRJEB31742, PRJNA629856, PRJNA737271 and PRJNA791695.
