# Supplementary material for: Metagenomics comparison identifies shared pathogenic microbiome in humans, pigs and chickens
Source: Appl Microbiol Biotechnol. 2026 Jul 8;110:292. doi: 10.1007/s00253-026-13948-1 (PMC13631169; doi:10.1007/s00253-026-13948-1)
Supplement: Supplementary file 1 [file 253_2026_13948_MOESM1_ESM.pdf]

Applied Microbiology and Biotechnology

Supplemental material

Metagenomics comparison identifies shared pathogenic microbiome in humans, pigs and chickens

Sung J. Yu[1], Dragana Stanley[1,2,*], Thi T. H. Van[1,3], Jason C. Steel[2,§], Yadav S. Bajagai[1,§]

[1]Institute for Future Farming Systems, Central Queensland University, Rockhampton, Queensland 4702, Australia

[2]School of Health, Medical and Applied Sciences, Central Queensland University, Rockhampton, Queensland 4702, Australia

[3]School of Science, RMIT University, Bundoora West Campus, Bundoora, VIC 3083, Australia

[§]These authors contributed equally to this work.

[*]Author to whom correspondence should be addressed.

Corresponding author: Professor Dragana Stanley, D.Stanley@cqu.edu.au

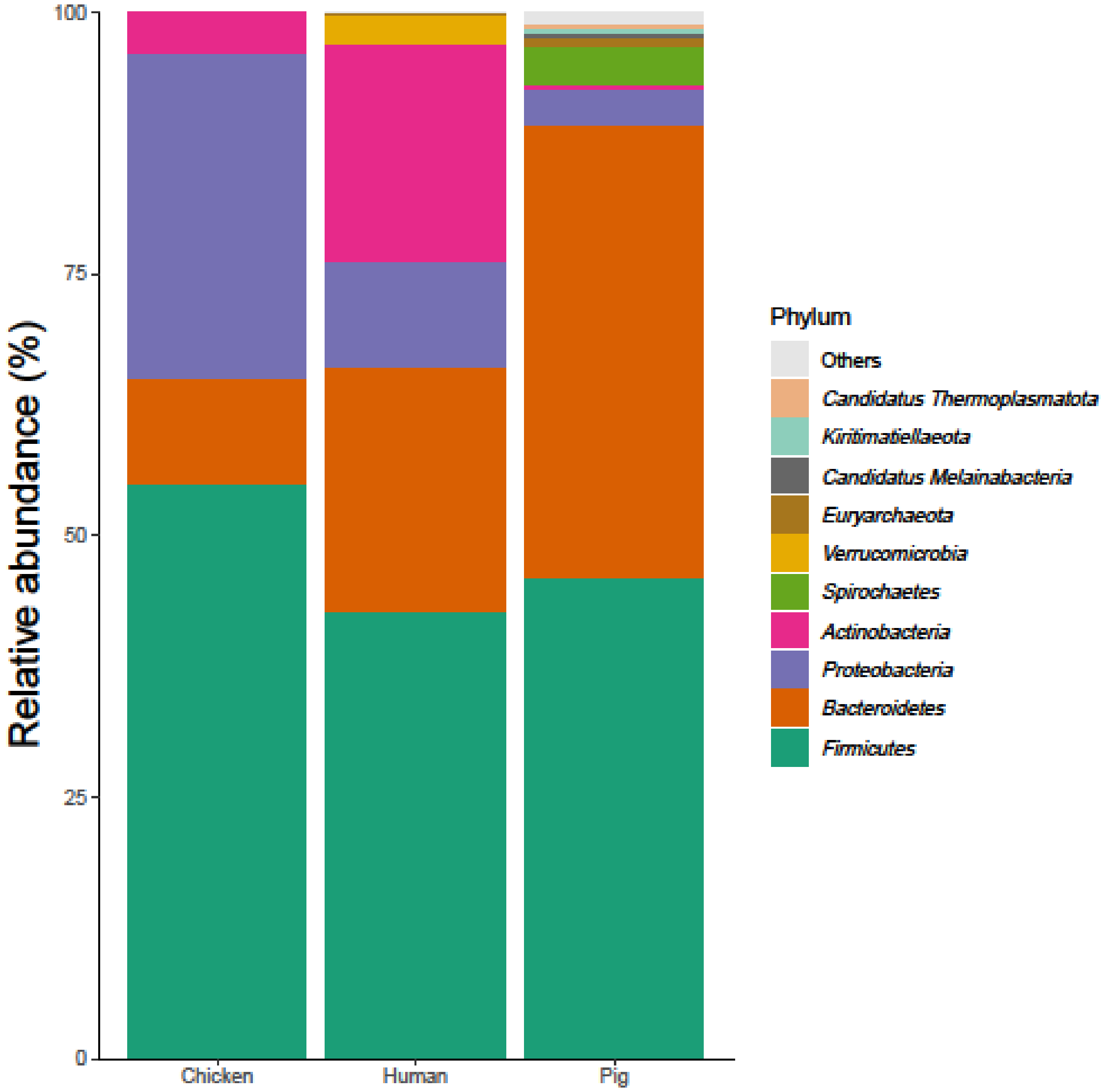

Supplementary Fig. S1: Relative abundance of the top 10 bacterial phyla identified in the faecal microbiota of chickens, humans, and pigs based on shotgun metagenomic sequencing. Bar plots represent the mean relative abundance (%) of phyla within each host group. Chicken samples were dominated mainly by *Firmicutes* and *Proteobacteria*, human samples showed a high relative abundance of *Firmicutes*, *Bacteroidetes* and *Actinobacteria*, and pig samples were characterised by *Firmicutes* and *Bacteroidetes*. Remaining low-abundance phyla were grouped as "Others".

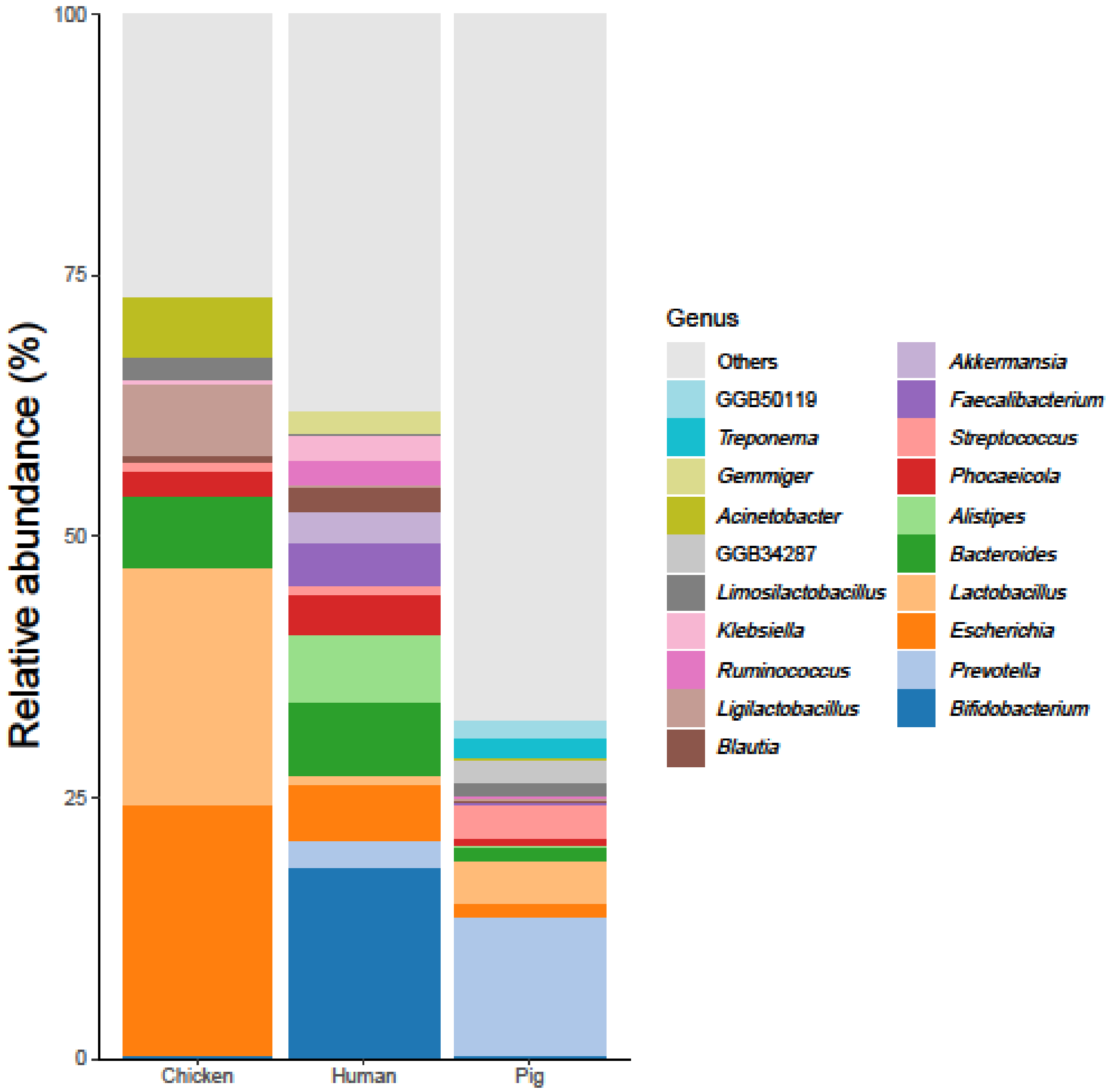

Supplementary Fig. S2: Relative abundance of the top 20 bacterial genera identified in the faecal microbiota of chickens, humans, and pigs based on shotgun metagenomic sequencing. Bar plots represent the mean relative abundance (%) of genera within each host group. Chickens showed higher abundances of *Escherichia* and *Lactobacillus*, humans were characterised by *Bifidobacterium*, *Bacteroides*, *Alistipes*, *Escherichia*, *Phocaeicola*, and *Faecalibacterium*, whereas pigs showed higher relative abundances of *Prevotella*, *Lactobacillus*, and *Streptococcus*. Remaining low-abundance genera were grouped as "Others".

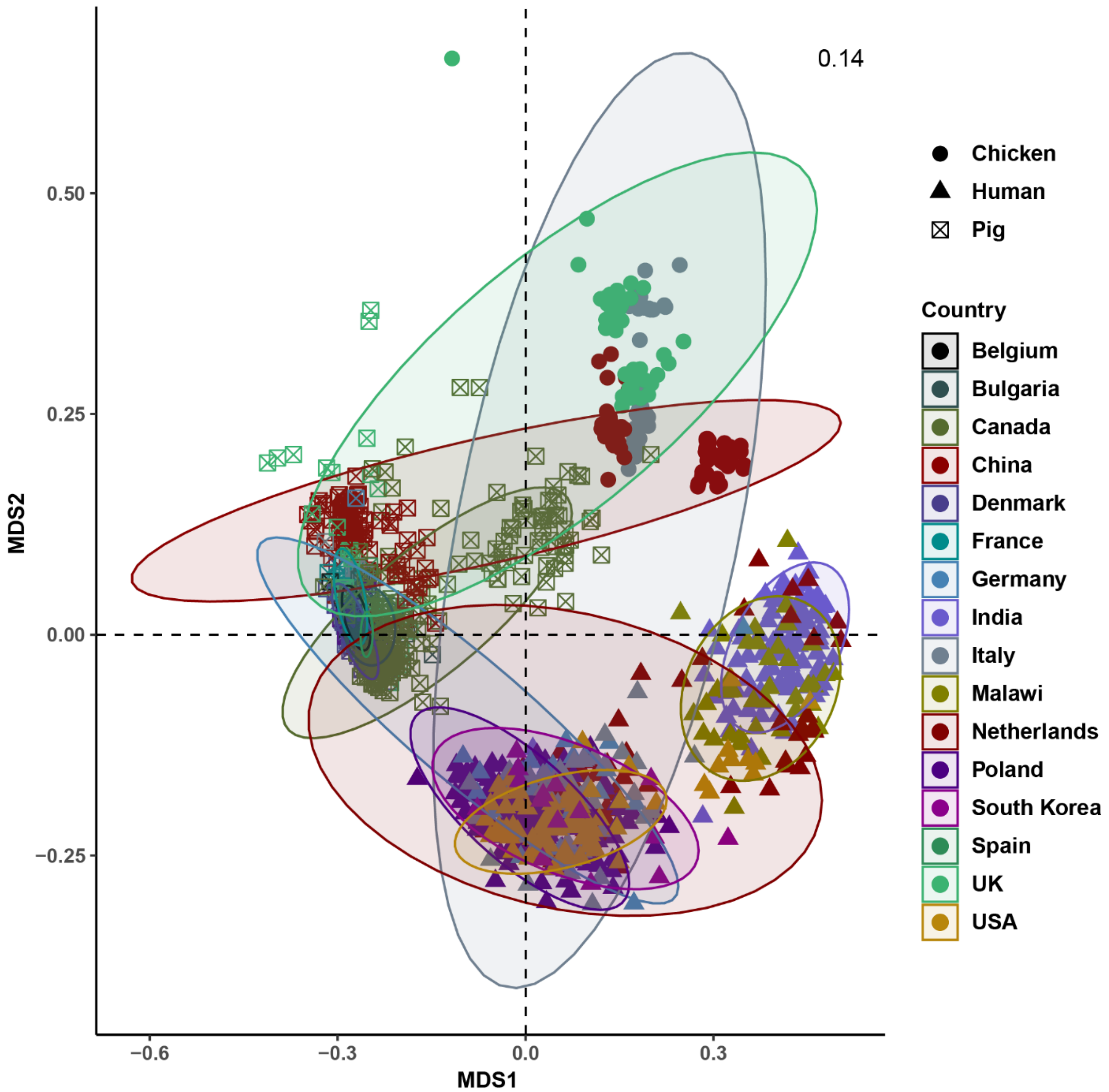

Supplementary Fig. S3: NMDS plot using Jaccard distance showing distribution and similarity of samples with symbols indicating the host species and colored by country of study.

Supplemntary Table S1: Sample-level accession number with number of quality filtered reads for each sample

Accession No.	Species	Country	No of quality filtered reads
ERR1135351	Pig	Denmark	7.42E+07
ERR1135352	Pig	Denmark	7.44E+07
ERR1135353	Pig	Denmark	8.99E+07
ERR1135355	Pig	Denmark	6.54E+07
ERR1135356	Pig	Denmark	6.86E+07
ERR1135357	Pig	Denmark	7.28E+07
ERR1135358	Pig	Denmark	6.92E+07
ERR1135359	Pig	Denmark	6.66E+07
ERR1135360	Pig	Denmark	6.74E+07
ERR1135361	Pig	Denmark	6.14E+07
ERR1135362	Pig	Denmark	7.26E+07
ERR1135363	Pig	Denmark	6.97E+07
ERR1135364	Pig	Denmark	8.06E+07
ERR1135365	Pig	Denmark	7.64E+07
ERR1135366	Pig	Denmark	5.99E+07
ERR1135367	Pig	Denmark	7.36E+07
ERR1135368	Pig	Denmark	7.73E+07
ERR1135369	Pig	Denmark	7.40E+07
ERR1135370	Pig	Denmark	6.32E+07
ERR1135371	Pig	Denmark	6.84E+07
ERR1135372	Pig	Denmark	7.25E+07
ERR1135373	Pig	Denmark	7.01E+07
ERR1135374	Pig	Denmark	7.44E+07
ERR1135375	Pig	Denmark	7.16E+07
ERR1135376	Pig	Denmark	7.24E+07
ERR1135377	Pig	Denmark	6.94E+07
ERR1135378	Pig	China	6.35E+07
ERR1135379	Pig	China	6.82E+07
ERR1135380	Pig	China	5.77E+07
ERR1135381	Pig	China	5.23E+07
ERR1135382	Pig	China	5.76E+07
ERR1135383	Pig	China	5.36E+07
ERR1135384	Pig	China	5.29E+07
ERR1135385	Pig	China	6.82E+07
ERR1135386	Pig	China	5.13E+07
ERR1135387	Pig	China	5.54E+07
ERR1135388	Pig	China	5.86E+07

ERR1135389	Pig	China	5.90E+07
ERR1135390	Pig	China	2.58E+07
ERR1135391	Pig	China	2.38E+07
ERR1135392	Pig	China	2.29E+07
ERR1135393	Pig	China	2.42E+07
ERR1135394	Pig	China	2.44E+07
ERR1135395	Pig	China	2.24E+07
ERR1135396	Pig	China	4.82E+07
ERR1135397	Pig	China	4.81E+07
ERR1135398	Pig	China	2.94E+07
ERR1135399	Pig	China	2.92E+07
ERR1135400	Pig	China	2.78E+07
ERR1135401	Pig	China	4.26E+07
ERR1135402	Pig	China	3.94E+07
ERR1135403	Pig	China	3.72E+07
ERR1135404	Pig	China	4.33E+07
ERR1135405	Pig	China	4.05E+07
ERR1135406	Pig	China	4.10E+07
ERR1135407	Pig	China	5.65E+07
ERR1135408	Pig	China	6.62E+07
ERR1135409	Pig	China	5.99E+07
ERR1135410	Pig	China	6.71E+07
ERR1135411	Pig	China	5.97E+07
ERR1135412	Pig	China	5.26E+07
ERR1135413	Pig	China	6.43E+07
ERR1135414	Pig	China	4.75E+07
ERR1135415	Pig	China	5.79E+07
ERR1135416	Pig	China	5.62E+07
ERR1135417	Pig	China	7.03E+07
ERR1135418	Pig	China	5.53E+07
ERR1135419	Pig	China	6.54E+07
ERR1135420	Pig	China	6.51E+07
ERR1135421	Pig	China	6.01E+07
ERR1135422	Pig	China	7.26E+07
ERR1135423	Pig	China	4.74E+07
ERR1135424	Pig	China	5.97E+07
ERR1135425	Pig	China	5.95E+07
ERR1135426	Pig	China	6.04E+07
ERR1135427	Pig	China	2.08E+07
ERR1135428	Pig	China	2.01E+07
ERR1135429	Pig	China	2.38E+07
ERR1135430	Pig	China	2.40E+07
ERR1135431	Pig	China	4.82E+07
ERR1135432	Pig	China	5.29E+07

ERR1135433	Pig	China	2.00E+07
ERR1135434	Pig	China	1.92E+07
ERR1135435	Pig	China	1.95E+07
ERR1135436	Pig	China	1.88E+07
ERR1135437	Pig	China	1.89E+07
ERR1135438	Pig	China	1.81E+07
ERR1135439	Pig	China	2.68E+07
ERR1135440	Pig	China	2.57E+07
ERR1135441	Pig	China	3.16E+07
ERR1135442	Pig	China	3.03E+07
ERR1135443	Pig	China	6.89E+07
ERR1135444	Pig	China	6.18E+07
ERR1135445	Pig	China	4.21E+07
ERR1135446	Pig	China	6.11E+07
ERR1135447	Pig	China	3.80E+07
ERR1135448	Pig	China	4.72E+07
ERR1135449	Pig	China	5.68E+07
ERR1135450	Pig	China	2.84E+07
ERR1135451	Pig	China	2.52E+07
ERR1135452	Pig	China	3.41E+07
ERR1135453	Pig	China	5.50E+07
ERR1135454	Pig	China	5.65E+07
ERR1135455	Pig	China	5.38E+07
ERR1135456	Pig	China	5.54E+07
ERR1135457	Pig	China	5.44E+07
ERR1135458	Pig	China	5.74E+07
ERR1135459	Pig	China	5.13E+07
ERR1135460	Pig	China	5.55E+07
ERR1135461	Pig	China	5.83E+07
ERR1135462	Pig	China	4.29E+07
ERR1135463	Pig	China	3.63E+07
ERR1135464	Pig	China	6.25E+07
ERR1135465	Pig	China	5.67E+07
ERR1135466	Pig	China	5.78E+07
ERR1135467	Pig	China	5.84E+07
ERR1135468	Pig	China	4.68E+07
ERR1135469	Pig	China	5.57E+07
ERR1135470	Pig	China	4.78E+07
ERR1135471	Pig	China	6.06E+07
ERR1135472	Pig	China	6.46E+07
ERR2026030	Chicken	Italy	1.50E+06
ERR2026031	Chicken	Italy	3.13E+06
ERR2026032	Chicken	Italy	4.38E+06
ERR2026033	Chicken	Italy	3.90E+06

ERR2026034	Chicken	Italy	8.92E+06
ERR2026035	Chicken	Italy	3.37E+06
ERR2026036	Chicken	Italy	3.24E+06
ERR2026037	Chicken	Italy	4.33E+06
ERR2026038	Chicken	Italy	3.90E+06
ERR2026039	Chicken	Italy	6.53E+06
ERR2026040	Chicken	Italy	6.44E+06
ERR2026041	Chicken	Italy	4.52E+06
ERR2026042	Chicken	Italy	4.13E+06
ERR2026046	Chicken	Italy	3.66E+06
ERR2026047	Chicken	Italy	6.23E+06
ERR2026369	Chicken	Italy	7.98E+06
ERR2026370	Chicken	Italy	1.70E+06
ERR2026371	Chicken	Italy	6.30E+06
ERR2026372	Chicken	Italy	5.21E+06
ERR2026373	Chicken	Italy	4.62E+06
ERR2026374	Chicken	Italy	8.67E+06
ERR2026375	Chicken	Italy	2.58E+06
ERR2026376	Chicken	Italy	4.50E+06
ERR2027011	Chicken	Italy	2.54E+06
ERR2027656	Chicken	Italy	3.55E+06
ERR2027657	Chicken	Italy	4.47E+06
ERR2027658	Chicken	Italy	7.18E+06
ERR2027659	Chicken	Italy	8.37E+06
ERR2027871	Chicken	Italy	5.33E+06
ERR2027872	Chicken	Italy	7.45E+06
ERR2027873	Chicken	Italy	5.85E+06
ERR2027874	Chicken	Italy	4.81E+06
ERR2027875	Chicken	Italy	3.95E+06
ERR2027876	Chicken	Italy	5.88E+06
ERR2027877	Chicken	Italy	4.17E+06
ERR2027878	Chicken	Italy	3.59E+06
ERR2027879	Chicken	Italy	2.42E+06
ERR2027880	Chicken	Italy	6.61E+06
ERR2028489	Chicken	Italy	5.31E+06
ERR2028490	Chicken	Italy	4.61E+06
ERR2028491	Chicken	Italy	5.61E+06
ERR2028492	Chicken	Italy	7.01E+06
ERR2028493	Chicken	Italy	3.60E+06
ERR2041806	Chicken	Italy	7.49E+06
ERR2044295	Chicken	Italy	4.84E+06
ERR2044296	Chicken	Italy	6.25E+06
ERR2044297	Chicken	Italy	7.00E+06
ERR2044634	Chicken	Italy	4.86E+06

ERR2044635	Chicken	Italy	6.84E+06
ERR2044636	Chicken	Italy	5.18E+06
ERR2044637	Chicken	Italy	6.64E+06
ERR2044638	Chicken	Italy	5.87E+06
ERR2044639	Chicken	Italy	5.16E+06
ERR2044640	Chicken	Italy	4.02E+06
ERR2044641	Chicken	Italy	3.84E+06
ERR2044642	Chicken	Italy	4.55E+06
ERR2044643	Chicken	Italy	4.22E+06
ERR2044644	Chicken	Italy	4.20E+06
ERR2044653	Chicken	Italy	4.60E+06
ERR2044654	Chicken	Italy	6.72E+06
ERR2044655	Chicken	Italy	4.06E+06
ERR2044656	Chicken	Italy	5.49E+06
ERR2241624	Pig	Netherlands	6.65E+07
ERR2241625	Pig	Netherlands	5.63E+07
ERR2241626	Pig	Netherlands	6.57E+07
ERR2241627	Pig	Netherlands	5.75E+07
ERR2241629	Pig	Netherlands	5.30E+07
ERR2241630	Pig	Netherlands	6.19E+07
ERR2241633	Pig	Netherlands	8.02E+07
ERR2241636	Pig	Netherlands	5.16E+07
ERR2241638	Pig	Netherlands	5.40E+07
ERR2241640	Pig	Netherlands	8.24E+07
ERR2241642	Pig	Netherlands	6.15E+07
ERR2241644	Pig	Netherlands	8.21E+07
ERR2241646	Pig	Netherlands	6.46E+07
ERR2241648	Pig	Netherlands	6.20E+07
ERR2241650	Pig	Netherlands	5.52E+07
ERR2241652	Pig	Netherlands	6.21E+07
ERR2241654	Pig	Netherlands	8.77E+07
ERR2241656	Pig	Netherlands	7.91E+07
ERR2241658	Pig	Netherlands	7.46E+07
ERR2241660	Pig	Netherlands	5.68E+07
ERR2241661	Pig	Netherlands	1.39E+08
ERR2241663	Pig	Netherlands	6.94E+07
ERR2241666	Pig	Germany	6.29E+07
ERR2241667	Pig	Germany	7.36E+07
ERR2241668	Pig	Germany	5.21E+07
ERR2241669	Pig	Germany	1.23E+08
ERR2241670	Pig	Germany	5.19E+07
ERR2241671	Pig	Germany	1.34E+08
ERR2241672	Pig	Germany	1.15E+08
ERR2241673	Pig	Germany	8.86E+07

ERR2241674	Pig	Germany	1.43E+08
ERR2241675	Pig	Germany	6.66E+07
ERR2241676	Pig	Germany	8.73E+07
ERR2241677	Pig	Germany	6.21E+07
ERR2241678	Pig	Germany	2.82E+07
ERR2241679	Pig	Germany	1.43E+08
ERR2241680	Pig	Germany	6.63E+07
ERR2241681	Pig	Germany	3.18E+06
ERR2241682	Pig	Germany	4.02E+07
ERR2241683	Pig	Germany	8.53E+07
ERR2241684	Pig	Germany	7.34E+07
ERR2241685	Pig	Germany	4.89E+07
ERR2241705	Pig	France	8.79E+07
ERR2241706	Pig	France	4.47E+07
ERR2241707	Pig	France	6.42E+07
ERR2241708	Pig	France	1.01E+08
ERR2241709	Pig	France	1.30E+07
ERR2241710	Pig	France	7.43E+07
ERR2241711	Pig	France	6.48E+07
ERR2241712	Pig	France	9.95E+07
ERR2241713	Pig	France	5.82E+07
ERR2241714	Pig	France	1.27E+08
ERR2241715	Pig	France	2.37E+07
ERR2241716	Pig	France	6.28E+07
ERR2241717	Pig	France	6.21E+07
ERR2241718	Pig	France	2.91E+07
ERR2241719	Pig	France	6.82E+07
ERR2241720	Pig	France	7.64E+07
ERR2241721	Pig	France	9.70E+07
ERR2241722	Pig	France	7.56E+07
ERR2241723	Pig	France	9.66E+07
ERR2241724	Pig	France	8.56E+07
ERR2241745	Pig	Spain	1.07E+08
ERR2241746	Pig	Spain	7.30E+07
ERR2241751	Pig	Spain	6.75E+07
ERR2241757	Pig	Spain	7.03E+07
ERR2241766	Pig	Spain	5.08E+07
ERR2241767	Pig	Spain	9.90E+07
ERR2241768	Pig	Spain	8.34E+07
ERR2241769	Pig	Spain	1.15E+08
ERR2241771	Pig	Spain	8.19E+07
ERR2241772	Pig	Spain	6.40E+07
ERR2241773	Pig	Spain	7.31E+07
ERR2241774	Pig	Spain	1.04E+08

ERR2241775	Pig	Spain	7.70E+07
ERR2241776	Pig	Spain	8.20E+07
ERR2241777	Pig	Spain	6.85E+07
ERR2241778	Pig	Spain	8.21E+07
ERR2241779	Pig	Spain	7.64E+07
ERR2241780	Pig	Spain	1.15E+08
ERR2241781	Pig	Spain	9.20E+07
ERR2241782	Pig	Spain	5.56E+07
ERR2241785	Pig	Belgium	5.98E+07
ERR2241786	Pig	Belgium	6.06E+07
ERR2241787	Pig	Belgium	6.17E+07
ERR2241789	Pig	Belgium	3.47E+07
ERR2241792	Pig	Belgium	3.19E+07
ERR2241793	Pig	Belgium	7.32E+07
ERR2241796	Pig	Belgium	5.56E+07
ERR2241797	Pig	Belgium	8.44E+07
ERR2241800	Pig	Belgium	6.31E+07
ERR2241804	Pig	Belgium	7.02E+07
ERR2241810	Pig	Belgium	4.55E+07
ERR2241811	Pig	Belgium	7.18E+07
ERR2241812	Pig	Belgium	6.94E+07
ERR2241813	Pig	Belgium	7.12E+07
ERR2241814	Pig	Belgium	2.85E+07
ERR2241816	Pig	Belgium	6.42E+07
ERR2241817	Pig	Belgium	7.12E+07
ERR2241820	Pig	Belgium	5.41E+07
ERR2241821	Pig	Belgium	5.61E+07
ERR2241822	Pig	Belgium	3.79E+07
ERR2241825	Pig	Belgium	4.59E+07
ERR2241826	Pig	Belgium	6.25E+07
ERR2241827	Pig	Italy	6.31E+07
ERR2241829	Pig	Italy	3.32E+07
ERR2241830	Pig	Italy	4.66E+07
ERR2241831	Pig	Italy	1.03E+08
ERR2241832	Pig	Italy	5.65E+07
ERR2241833	Pig	Italy	3.88E+07
ERR2241834	Pig	Italy	6.31E+07
ERR2241835	Pig	Italy	5.95E+07
ERR2241836	Pig	Italy	3.89E+07
ERR2241837	Pig	Italy	4.23E+07
ERR2241838	Pig	Italy	4.77E+07
ERR2241839	Pig	Italy	5.49E+07
ERR2241840	Pig	Italy	5.92E+07
ERR2241841	Pig	Italy	8.42E+07

ERR2241842	Pig	Italy	5.38E+07
ERR2241843	Pig	Italy	6.40E+07
ERR2241844	Pig	Italy	5.80E+07
ERR2241845	Pig	Italy	5.65E+07
ERR2241846	Pig	Italy	6.02E+07
ERR2241847	Pig	Italy	7.23E+07
ERR2241867	Pig	Poland	5.55E+07
ERR2241868	Pig	Poland	4.94E+07
ERR2241869	Pig	Poland	6.40E+07
ERR2241870	Pig	Poland	7.91E+07
ERR2241871	Pig	Poland	4.03E+07
ERR2241872	Pig	Poland	2.43E+07
ERR2241873	Pig	Poland	4.68E+07
ERR2241874	Pig	Poland	5.87E+07
ERR2241875	Pig	Poland	6.13E+07
ERR2241876	Pig	Poland	5.83E+07
ERR2241877	Pig	Poland	4.34E+07
ERR2241878	Pig	Poland	6.33E+07
ERR2241879	Pig	Poland	6.36E+07
ERR2241880	Pig	Poland	5.99E+07
ERR2241881	Pig	Poland	6.45E+07
ERR2241882	Pig	Poland	6.32E+07
ERR2241883	Pig	Poland	5.92E+07
ERR2241884	Pig	Poland	6.17E+07
ERR2241885	Pig	Poland	8.65E+07
ERR2241886	Pig	Poland	8.01E+07
ERR2241908	Pig	Denmark	7.12E+07
ERR2241910	Pig	Denmark	8.10E+07
ERR2241912	Pig	Denmark	5.55E+07
ERR2241914	Pig	Denmark	7.08E+07
ERR2241916	Pig	Denmark	1.17E+08
ERR2241918	Pig	Denmark	1.02E+08
ERR2241920	Pig	Denmark	1.13E+08
ERR2241922	Pig	Denmark	6.21E+07
ERR2241924	Pig	Denmark	8.30E+07
ERR2241926	Pig	Denmark	5.14E+07
ERR2241928	Pig	Denmark	6.04E+07
ERR2241930	Pig	Denmark	7.09E+07
ERR2241932	Pig	Denmark	9.99E+07
ERR2241934	Pig	Denmark	9.82E+07
ERR2241936	Pig	Denmark	1.60E+08
ERR2241938	Pig	Denmark	9.26E+07
ERR2241940	Pig	Denmark	9.59E+07
ERR2241942	Pig	Denmark	8.18E+07

ERR2241944	Pig	Denmark	9.05E+07
ERR2241946	Pig	Denmark	7.65E+07
ERR2241947	Pig	Bulgaria	4.32E+07
ERR2241948	Pig	Bulgaria	8.11E+07
ERR2241949	Pig	Bulgaria	5.52E+07
ERR2241951	Pig	Bulgaria	3.98E+07
ERR2241958	Pig	Bulgaria	2.08E+07
ERR2241959	Pig	Bulgaria	5.84E+07
ERR2241960	Pig	Bulgaria	3.37E+07
ERR2241961	Pig	Bulgaria	5.79E+07
ERR2241962	Pig	Bulgaria	6.27E+07
ERR2241963	Pig	Bulgaria	4.35E+07
ERR2241964	Pig	Bulgaria	3.15E+07
ERR2241965	Pig	Bulgaria	3.96E+07
ERR2241966	Pig	Bulgaria	4.75E+07
ERR2241967	Pig	Bulgaria	4.98E+07
ERR2241968	Pig	Bulgaria	9.81E+07
ERR2241969	Pig	Bulgaria	2.79E+07
ERR2241970	Pig	Bulgaria	3.54E+07
ERR2241973	Pig	Bulgaria	2.82E+07
ERR2241974	Pig	Bulgaria	1.45E+07
ERR2241975	Pig	Bulgaria	2.01E+07
ERR2241976	Pig	Bulgaria	7.96E+06
ERR3588729	Pig	UK	9.95E+06
ERR3588730	Pig	UK	9.92E+06
ERR3588731	Pig	UK	9.74E+06
ERR3588732	Pig	UK	9.97E+06
ERR3588733	Pig	UK	9.74E+06
ERR3588734	Pig	UK	9.22E+06
ERR3588735	Pig	UK	7.80E+06
ERR3588736	Pig	UK	8.72E+06
ERR3588737	Pig	UK	8.04E+06
ERR3588738	Pig	UK	8.78E+06
ERR3588739	Pig	UK	9.74E+06
ERR3588740	Pig	UK	1.02E+07
ERR3588741	Pig	UK	1.03E+07
ERR3588742	Pig	UK	7.33E+06
ERR3588743	Pig	UK	8.29E+06
ERR3588744	Pig	UK	8.68E+06
ERR3588745	Pig	UK	8.12E+06
ERR3588746	Pig	UK	1.10E+07
ERR3588747	Pig	UK	9.93E+06
ERR3588749	Pig	UK	1.01E+07
ERR3588750	Pig	UK	1.01E+07

ERR3588751	Pig	UK	9.68E+06
ERR3588752	Pig	UK	1.05E+07
ERR3588753	Pig	UK	1.01E+07
ERR3588754	Pig	UK	9.52E+06
ERR3588755	Pig	UK	9.93E+06
ERR3648472	Pig	China	1.31E+08
ERR3648605	Pig	China	1.07E+08
ERR3648606	Pig	China	1.39E+08
ERR11438005	Human	India	3.26E+07
ERR11438014	Human	India	2.68E+07
ERR11438035	Human	India	2.00E+07
ERR11438040	Human	India	1.49E+07
ERR11438041	Human	India	2.86E+07
ERR11438043	Human	India	3.13E+07
ERR11438046	Human	India	1.55E+07
ERR11438048	Human	India	4.01E+07
ERR11438049	Human	India	2.81E+07
ERR11438053	Human	India	1.46E+07
ERR11438054	Human	India	1.57E+07
ERR11438056	Human	India	1.73E+07
ERR11438057	Human	India	3.72E+07
ERR11438063	Human	India	3.65E+07
ERR11438064	Human	India	1.47E+07
ERR11438065	Human	India	9.64E+06
ERR11438069	Human	India	2.65E+07
ERR11438079	Human	India	1.27E+07
ERR11438080	Human	India	1.36E+07
ERR11438085	Human	India	2.32E+07
ERR11438086	Human	India	2.05E+07
ERR11438089	Human	India	3.59E+07
ERR11438090	Human	India	3.46E+07
ERR11438111	Human	India	3.87E+07
ERR11438113	Human	India	1.64E+07
ERR11438186	Human	India	2.71E+07
ERR11438190	Human	India	1.93E+07
ERR11438194	Human	India	4.42E+07
ERR11438195	Human	India	4.42E+07
ERR11438197	Human	India	3.17E+07
ERR11438198	Human	India	3.72E+07
ERR11438200	Human	India	1.36E+07
ERR11438207	Human	India	1.95E+07
ERR11438208	Human	India	5.05E+07
ERR11438209	Human	India	4.14E+07
ERR11438212	Human	India	2.21E+07

ERR11438214	Human	India	1.40E+07
ERR11438216	Human	India	1.78E+07
ERR11438221	Human	India	2.60E+07
ERR11438238	Human	India	1.64E+07
ERR11438242	Human	India	2.96E+07
ERR11438244	Human	India	1.35E+07
ERR11438248	Human	India	1.19E+07
ERR11438253	Human	India	2.76E+07
ERR11438254	Human	India	3.52E+07
ERR11438256	Human	India	2.74E+07
ERR11438263	Human	India	1.37E+07
ERR11438271	Human	India	2.06E+07
ERR11438273	Human	India	2.84E+07
ERR11438274	Human	India	3.29E+07
ERR11438280	Human	India	2.02E+07
ERR11438281	Human	India	1.01E+07
ERR11438282	Human	India	1.64E+07
ERR11438285	Human	India	8.10E+06
ERR11438286	Human	India	1.20E+07
ERR11438288	Human	India	3.94E+07
ERR11438289	Human	India	9.70E+06
ERR11438290	Human	India	3.35E+07
ERR11438291	Human	India	7.20E+06
ERR11438295	Human	India	1.29E+07
ERR11438296	Human	India	2.31E+07
ERR11438297	Human	India	2.78E+07
ERR11438302	Human	India	2.89E+07
ERR11438305	Human	India	2.29E+07
ERR11438404	Human	India	3.09E+07
ERR11438406	Human	India	2.18E+07
ERR11438411	Human	India	1.92E+07
ERR11438433	Human	India	5.13E+07
ERR11438846	Human	India	3.92E+07
ERR11438893	Human	India	6.31E+06
ERR11438912	Human	India	2.55E+07
ERR11439067	Human	India	1.78E+07
ERR11439092	Human	India	1.43E+07
ERR11439095	Human	India	2.99E+07
ERR11439096	Human	India	3.32E+07
ERR11439140	Human	India	2.74E+07
ERR11439273	Human	India	2.62E+07
ERR11439284	Human	India	5.42E+06
ERR11439591	Human	India	3.00E+07
ERR11439599	Human	India	1.02E+07

ERR11439660	Human	India	1.41E+07
ERR11439695	Human	India	2.64E+07
ERR11439706	Human	India	4.23E+07
ERR11439710	Human	India	3.06E+07
ERR11439714	Human	India	2.30E+07
ERR11440740	Human	India	1.77E+07
ERR11440753	Human	India	5.55E+07
ERR11440797	Human	India	3.30E+07
ERR11441010	Human	India	2.46E+07
ERR11441076	Human	India	1.12E+07
ERR11441222	Human	India	3.95E+07
ERR11441229	Human	India	9.39E+06
ERR11441247	Human	India	2.97E+07
ERR11441256	Human	India	3.80E+07
ERR11441272	Human	Malawi	2.30E+07
ERR11441273	Human	Malawi	9.36E+06
ERR11441305	Human	Malawi	9.90E+06
ERR11441306	Human	Malawi	2.39E+07
ERR11441308	Human	Malawi	2.92E+07
ERR11441547	Human	Malawi	1.07E+07
ERR11441548	Human	Malawi	8.89E+06
ERR11441551	Human	Malawi	2.28E+07
ERR11441553	Human	Malawi	1.42E+07
ERR11441554	Human	Malawi	3.80E+07
ERR11441555	Human	Malawi	1.04E+07
ERR11441557	Human	Malawi	2.33E+07
ERR11441697	Human	Malawi	4.76E+07
ERR11441699	Human	Malawi	1.71E+07
ERR11441702	Human	Malawi	3.30E+07
ERR11441704	Human	Malawi	1.83E+07
ERR11441708	Human	Malawi	1.03E+07
ERR11441710	Human	Malawi	3.76E+07
ERR11441711	Human	Malawi	2.06E+07
ERR11441716	Human	Malawi	5.35E+06
ERR11441717	Human	Malawi	1.56E+07
ERR11441718	Human	Malawi	7.57E+06
ERR11441719	Human	Malawi	2.20E+07
ERR11441721	Human	Malawi	2.15E+07
ERR11441724	Human	Malawi	2.53E+07
ERR11441937	Human	Malawi	2.10E+07
ERR11441939	Human	Malawi	2.51E+07
ERR11441941	Human	Malawi	1.48E+07
ERR11441942	Human	Malawi	9.95E+06
ERR11441943	Human	Malawi	1.31E+07

ERR11441946	Human	Malawi	2.95E+07
ERR11442108	Human	Malawi	4.40E+07
ERR11442109	Human	Malawi	9.35E+06
ERR11442110	Human	Malawi	3.94E+07
ERR11442677	Human	Malawi	3.40E+07
ERR11442678	Human	Malawi	1.14E+07
ERR11442679	Human	Malawi	1.81E+07
ERR11442680	Human	Malawi	2.00E+07
ERR11442682	Human	Malawi	1.36E+07
ERR11820447	Human	Netherlands	5.94E+07
ERR11830072	Human	Netherlands	6.71E+07
ERR11834350	Human	Netherlands	6.47E+07
ERR11834358	Human	Netherlands	9.96E+07
ERR11834365	Human	Netherlands	1.13E+08
ERR11834368	Human	Netherlands	5.93E+07
ERR11834446	Human	Netherlands	7.93E+07
ERR11834471	Human	Netherlands	8.50E+07
ERR11834476	Human	Netherlands	7.46E+07
ERR11834480	Human	Netherlands	6.61E+07
ERR11834486	Human	Netherlands	6.57E+07
ERR11834491	Human	Netherlands	7.34E+07
ERR11834500	Human	Netherlands	1.42E+08
ERR11834505	Human	Netherlands	7.74E+07
ERR11834510	Human	Netherlands	6.63E+07
ERR11834516	Human	Netherlands	6.01E+07
ERR11834520	Human	Netherlands	6.08E+07
ERR11834683	Human	Netherlands	8.78E+07
ERR11834802	Human	Netherlands	6.30E+07
ERR11834806	Human	Netherlands	7.74E+07
ERR11834810	Human	Netherlands	6.27E+07
ERR11834821	Human	Netherlands	9.56E+07
ERR11834825	Human	Netherlands	7.15E+07
ERR11834835	Human	Netherlands	9.49E+07
ERR11834846	Human	Netherlands	9.11E+07
ERR11834853	Human	Netherlands	8.18E+07
ERR11834862	Human	Netherlands	1.05E+08
ERR11834878	Human	Netherlands	2.64E+08
ERR11834883	Human	Netherlands	6.52E+07
ERR11834894	Human	Netherlands	1.10E+08
ERR11834948	Human	Netherlands	7.85E+07
ERR11835515	Human	Netherlands	2.27E+08
ERR11835539	Human	Netherlands	5.79E+08
ERR11835548	Human	Netherlands	7.69E+07
ERR11835553	Human	Netherlands	1.22E+08

ERR11835557	Human	Netherlands	8.58E+07
ERR11835565	Human	Netherlands	6.17E+07
ERR11835572	Human	Netherlands	6.31E+07
ERR11835577	Human	Netherlands	6.43E+07
ERR11835582	Human	Netherlands	6.03E+07
ERR11835593	Human	Netherlands	1.02E+08
ERR11835596	Human	Netherlands	6.46E+07
ERR11835602	Human	Netherlands	6.18E+07
ERR11835605	Human	Netherlands	6.80E+07
ERR11835613	Human	Netherlands	1.02E+08
ERR11835627	Human	Netherlands	7.11E+07
ERR11835632	Human	Netherlands	8.06E+07
ERR11835640	Human	Netherlands	1.10E+08
ERR11835645	Human	Netherlands	6.56E+07
ERR11835742	Human	Netherlands	9.45E+07
ERR11835750	Human	Netherlands	6.53E+07
ERR11835756	Human	Netherlands	7.00E+07
ERR11835764	Human	Netherlands	6.64E+07
ERR11836200	Human	Netherlands	1.78E+08
ERR11836206	Human	Netherlands	8.59E+07
ERR11836216	Human	Netherlands	6.10E+07
ERR11836228	Human	Netherlands	1.72E+08
ERR11836230	Human	Netherlands	7.63E+07
ERR11836238	Human	Netherlands	1.25E+08
ERR11836687	Human	Netherlands	3.62E+08
ERR11836692	Human	Netherlands	9.89E+07
ERR11836699	Human	Netherlands	8.99E+07
ERR11836707	Human	Netherlands	9.05E+07
ERR11836711	Human	Netherlands	1.00E+08
ERR11836715	Human	Netherlands	7.31E+07
ERR11836722	Human	Netherlands	1.08E+08
ERR11836726	Human	Netherlands	6.17E+07
ERR11836842	Human	Netherlands	6.60E+07
ERR11836849	Human	Netherlands	8.74E+07
ERR11836855	Human	Netherlands	6.45E+07
ERR11836863	Human	Netherlands	8.57E+07
ERR11836866	Human	Netherlands	4.14E+07
ERR11836876	Human	Netherlands	9.20E+07
ERR11836881	Human	Netherlands	7.91E+07
ERR11836885	Human	Netherlands	8.15E+07
ERR11836901	Human	Netherlands	6.65E+07
ERR11836910	Human	Netherlands	8.55E+07
ERR11836920	Human	Netherlands	9.61E+07
ERR11836930	Human	Netherlands	1.33E+08

ERR11836940	Human	Netherlands	1.11E+08
ERR11836944	Human	Netherlands	7.12E+07
SRR25951014	Human	Poland	3.35E+07
SRR25951015	Human	Poland	4.08E+07
SRR25951016	Human	Poland	4.36E+07
SRR25951017	Human	Poland	1.85E+07
SRR25951018	Human	Poland	4.13E+07
SRR25951019	Human	Poland	4.92E+07
SRR25951020	Human	Poland	4.51E+07
SRR25951021	Human	Poland	4.51E+07
SRR25951022	Human	Poland	3.97E+07
SRR25951023	Human	Poland	5.14E+07
SRR25951024	Human	Poland	5.35E+07
SRR25951025	Human	Poland	4.00E+07
SRR25951026	Human	Poland	5.18E+07
SRR25951027	Human	Poland	4.72E+07
SRR25951028	Human	Poland	4.83E+07
SRR25951029	Human	Poland	5.03E+07
SRR25951030	Human	Poland	4.59E+07
SRR25951031	Human	Poland	3.26E+07
SRR25951032	Human	Poland	4.67E+07
SRR25951033	Human	Poland	4.52E+07
SRR25951034	Human	Poland	5.07E+07
SRR25951035	Human	Poland	4.35E+07
SRR25951036	Human	Poland	2.52E+07
SRR25951037	Human	Poland	4.42E+07
SRR25951038	Human	Poland	3.42E+07
SRR25951039	Human	Poland	4.17E+07
SRR25951040	Human	Poland	4.09E+07
SRR25951041	Human	Poland	3.83E+07
SRR25951042	Human	Poland	4.66E+07
SRR25951043	Human	Poland	4.65E+07
SRR25951044	Human	Poland	4.88E+07
SRR25951045	Human	Poland	4.10E+07
SRR25951046	Human	Poland	3.56E+07
SRR25951047	Human	Poland	4.39E+07
SRR25951048	Human	Poland	4.52E+07
SRR25951049	Human	Poland	3.77E+07
SRR25951050	Human	Poland	4.32E+07
SRR25951051	Human	Poland	3.17E+07
SRR25951052	Human	Poland	4.91E+07
SRR25951053	Human	Poland	4.46E+07
SRR25951054	Human	Poland	4.16E+07
SRR25951055	Human	Poland	2.54E+07

SRR25951056	Human	Poland	4.70E+07
SRR25951057	Human	Poland	3.96E+07
SRR25951058	Human	Poland	2.11E+07
SRR25951059	Human	Poland	8.55E+07
SRR25951060	Human	Poland	9.80E+07
SRR25951061	Human	Poland	8.12E+07
SRR25951062	Human	Poland	5.02E+07
SRR25951063	Human	Poland	3.43E+07
SRR25951064	Human	Poland	3.29E+07
SRR25951065	Human	Poland	9.85E+07
SRR25951066	Human	Poland	3.83E+07
SRR25951067	Human	Poland	9.12E+07
SRR25951068	Human	Poland	9.83E+07
SRR25951069	Human	Poland	2.31E+07
SRR25951070	Human	Poland	9.82E+07
SRR25951071	Human	Poland	4.48E+07
SRR25951072	Human	Poland	9.86E+07
SRR25951073	Human	Poland	4.89E+07
SRR25951074	Human	Poland	9.85E+07
SRR25951075	Human	Poland	2.94E+07
SRR25951076	Human	Poland	4.42E+07
SRR25951077	Human	Poland	3.99E+07
SRR25951078	Human	Poland	3.52E+07
SRR25951079	Human	Poland	4.06E+07
SRR25951080	Human	Poland	4.12E+07
SRR25951081	Human	Poland	3.71E+07
SRR27176114	Human	Poland	2.02E+07
SRR27176115	Human	Poland	2.11E+07
SRR27176116	Human	Poland	3.02E+07
SRR27176117	Human	Poland	2.49E+07
SRR27176118	Human	Poland	3.65E+07
SRR27176119	Human	Poland	2.29E+07
SRR27176120	Human	Poland	2.39E+07
SRR27176121	Human	Poland	2.36E+07
SRR27176122	Human	Poland	2.23E+07
SRR27176123	Human	Poland	3.02E+07
SRR27176124	Human	Poland	3.18E+07
SRR27176125	Human	Poland	2.52E+07
SRR27176126	Human	Poland	2.28E+07
SRR27176127	Human	Poland	2.71E+07
SRR27176128	Human	Poland	3.09E+07
SRR27176129	Human	Poland	2.08E+07
SRR27176130	Human	Poland	2.70E+07
SRR27176131	Human	Poland	2.80E+07

SRR27176132	Human	Poland	3.13E+07
SRR27176133	Human	Poland	2.64E+07
SRR27176134	Human	Poland	3.36E+07
SRR27176135	Human	Poland	2.71E+07
SRR27176136	Human	Poland	2.94E+07
SRR27176137	Human	Poland	2.61E+07
SRR27176138	Human	Poland	3.86E+07
SRR27176139	Human	Poland	3.24E+07
SRR27176140	Human	Poland	2.92E+07
SRR27176141	Human	Poland	2.92E+07
SRR27176142	Human	Poland	3.89E+07
SRR27176143	Human	Poland	2.11E+07
SRR27176144	Human	Poland	3.50E+07
SRR27176145	Human	Poland	4.44E+07
SRR27176146	Human	Poland	5.77E+07
SRR27176147	Human	Poland	5.41E+07
SRR27176148	Human	Poland	5.16E+07
SRR27176149	Human	Poland	4.25E+07
SRR27176150	Human	Poland	4.94E+07
SRR27176151	Human	Poland	4.88E+07
SRR27176152	Human	Poland	2.87E+07
SRR27176153	Human	Poland	3.96E+07
SRR27176154	Human	Poland	5.28E+07
SRR27176155	Human	Poland	3.90E+07
SRR27176156	Human	Poland	4.52E+07
SRR27176157	Human	Poland	3.92E+07
SRR27176158	Human	Poland	2.81E+07
SRR27176159	Human	Poland	3.88E+07
SRR27176160	Human	Poland	4.57E+07
SRR27176161	Human	Poland	3.19E+07
SRR27176162	Human	Poland	4.19E+07
SRR27176163	Human	Poland	2.37E+07
SRR27176164	Human	Poland	3.67E+07
SRR27176165	Human	Poland	4.14E+07
SRR27176167	Human	Poland	3.12E+07
SRR27176168	Human	Poland	3.85E+07
SRR27176169	Human	Poland	3.56E+07
SRR27176170	Human	Poland	2.84E+07
SRR27176171	Human	Poland	3.36E+07
SRR27176172	Human	Poland	2.70E+07
SRR27176173	Human	Poland	3.64E+07
SRR27176174	Human	Poland	2.52E+07
SRR27176175	Human	Poland	2.15E+07
SRR27445723	Human	Poland	3.65E+07

SRR27445724	Human	Poland	2.74E+07
SRR27445725	Human	Poland	3.18E+07
SRR27445726	Human	Poland	2.77E+07
SRR27445727	Human	Poland	2.36E+07
SRR27445728	Human	Poland	2.34E+07
SRR27445729	Human	Poland	2.86E+07
SRR27445730	Human	Poland	3.18E+07
SRR27445731	Human	Poland	3.37E+07
SRR27445732	Human	Poland	2.42E+07
SRR27445733	Human	Poland	2.75E+07
SRR27445734	Human	Poland	2.50E+07
SRR27445735	Human	Poland	2.57E+07
SRR27445736	Human	Poland	5.01E+07
SRR27445737	Human	Poland	5.25E+07
SRR27445738	Human	Poland	5.46E+07
SRR27445739	Human	Poland	3.36E+07
SRR27445740	Human	Poland	2.90E+07
SRR27445741	Human	Poland	2.73E+07
SRR27445742	Human	Poland	2.64E+07
SRR27445743	Human	Poland	3.79E+07
SRR27445744	Human	Poland	2.59E+07
SRR27445745	Human	Poland	2.97E+07
SRR25662876	Human	USA	4.09E+07
SRR25662879	Human	USA	5.07E+07
SRR25662881	Human	USA	6.45E+07
SRR25662885	Human	USA	3.19E+07
SRR25662897	Human	USA	5.32E+07
SRR25662911	Human	USA	3.02E+07
SRR25662914	Human	USA	1.43E+07
SRR25662916	Human	USA	1.89E+07
SRR25662918	Human	USA	1.35E+07
SRR25662919	Human	USA	2.95E+06
SRR25825493	Human	Germany	4.85E+07
SRR25825494	Human	Germany	6.60E+07
SRR25825495	Human	Germany	4.91E+07
SRR25825496	Human	Germany	4.94E+07
SRR25825497	Human	Germany	6.04E+07
SRR25825498	Human	Germany	4.72E+07
SRR25825499	Human	Germany	5.07E+07
SRR25825500	Human	Germany	5.58E+07
SRR25825501	Human	Germany	4.52E+07
SRR25825502	Human	Germany	5.90E+07
SRR25825503	Human	Germany	6.93E+07
SRR25825504	Human	Germany	7.12E+07

SRR25825505	Human	Germany	5.58E+07
SRR25825506	Human	Germany	4.95E+07
SRR25825507	Human	Germany	4.77E+07
SRR25825508	Human	Germany	4.48E+07
SRR25825509	Human	Germany	5.76E+07
SRR25825510	Human	Germany	6.40E+07
SRR25825511	Human	Germany	6.23E+07
SRR25825512	Human	Germany	4.28E+07
SRR25825513	Human	Germany	5.12E+07
SRR25825514	Human	Germany	4.21E+07
SRR25825515	Human	Germany	4.51E+07
SRR25825516	Human	Germany	5.99E+07
SRR25825517	Human	Germany	5.65E+07
SRR25825518	Human	Germany	4.93E+07
SRR25825519	Human	Germany	6.08E+07
SRR25825520	Human	Germany	6.02E+07
SRR25825521	Human	Germany	5.40E+07
SRR26700564	Human	Italy	2.85E+07
SRR26700565	Human	Italy	2.51E+07
SRR26700566	Human	Italy	2.45E+07
SRR26700567	Human	Italy	2.95E+07
SRR26700568	Human	Italy	1.76E+07
SRR26700569	Human	Italy	1.95E+07
SRR26700570	Human	Italy	2.75E+07
SRR26700571	Human	Italy	1.79E+07
SRR26700572	Human	Italy	1.50E+07
SRR26700573	Human	Italy	3.52E+07
SRR26700574	Human	Italy	2.10E+07
SRR26700575	Human	Italy	5.71E+07
SRR26700576	Human	Italy	1.98E+07
SRR26700577	Human	Italy	2.09E+07
SRR26700578	Human	Italy	2.83E+07
SRR26700579	Human	Italy	1.62E+07
SRR26700580	Human	Italy	2.89E+07
SRR26700581	Human	Italy	2.47E+07
SRR26700582	Human	Italy	2.81E+07
SRR26700583	Human	Italy	3.45E+07
SRR26700584	Human	Italy	1.18E+07
SRR26700585	Human	Italy	4.54E+07
SRR26700586	Human	Italy	2.32E+07
SRR26700587	Human	Italy	1.17E+07
SRR26700588	Human	Italy	2.93E+07
SRR26700589	Human	Italy	1.23E+07
SRR26700590	Human	Italy	2.06E+07

SRR26700591	Human	Italy	2.95E+07
SRR26700592	Human	Italy	9.27E+06
SRR26700593	Human	Italy	1.15E+07
SRR26700594	Human	Italy	2.14E+07
SRR26700595	Human	Italy	4.67E+07
SRR26700596	Human	Italy	3.70E+07
SRR26700597	Human	Italy	1.80E+07
SRR26700598	Human	Italy	1.11E+07
SRR26700599	Human	Italy	8.47E+06
SRR26700600	Human	Italy	2.44E+07
SRR26700601	Human	Italy	2.66E+07
SRR26700602	Human	Italy	3.61E+07
SRR26700603	Human	Italy	3.13E+07
SRR26700604	Human	Italy	1.33E+07
SRR26700605	Human	Italy	1.53E+07
SRR26700606	Human	Italy	3.23E+07
SRR26700607	Human	Italy	2.51E+07
SRR27117388	Human	USA	9.93E+07
SRR27117389	Human	USA	1.09E+08
SRR27117390	Human	USA	1.06E+08
SRR27117391	Human	USA	8.15E+07
SRR27117392	Human	USA	1.05E+08
SRR27117393	Human	USA	1.17E+08
SRR27117394	Human	USA	1.28E+08
SRR27117395	Human	USA	1.24E+08
SRR27117396	Human	USA	1.08E+08
SRR27117397	Human	USA	2.00E+08
SRR27117398	Human	USA	1.05E+08
SRR27117399	Human	USA	1.15E+08
SRR27117400	Human	USA	1.18E+08
SRR27117401	Human	USA	1.23E+08
SRR27117402	Human	USA	1.01E+08
SRR27117403	Human	USA	1.24E+08
SRR27117404	Human	USA	1.02E+08
SRR27117405	Human	USA	1.22E+08
SRR27117406	Human	USA	1.05E+08
SRR27117407	Human	USA	1.05E+08
SRR27117408	Human	USA	9.42E+07
SRR27117409	Human	USA	1.11E+08
SRR27117410	Human	USA	8.19E+07
SRR27117411	Human	USA	1.25E+08
SRR27117412	Human	USA	9.42E+07
SRR27117413	Human	USA	1.14E+08
SRR27117414	Human	USA	1.11E+08

SRR27117415	Human	USA	6.37E+07
SRR27117416	Human	USA	1.05E+08
SRR27117417	Human	USA	1.25E+08
SRR27117418	Human	USA	8.73E+07
SRR27117419	Human	USA	9.68E+07
SRR27117420	Human	USA	1.04E+08
SRR27117421	Human	USA	1.05E+08
SRR27117422	Human	USA	9.79E+07
SRR27117423	Human	USA	1.12E+08
SRR27117424	Human	USA	9.71E+07
SRR27117425	Human	USA	1.22E+08
SRR27117426	Human	USA	1.19E+08
SRR27117427	Human	USA	1.81E+08
SRR27117428	Human	USA	1.08E+08
SRR27117429	Human	USA	1.14E+08
SRR27117430	Human	USA	1.13E+08
SRR27117431	Human	USA	1.34E+08
SRR27117432	Human	USA	9.92E+07
SRR27117433	Human	USA	1.05E+08
SRR27117434	Human	USA	1.01E+08
SRR27117435	Human	USA	8.81E+07
SRR27117436	Human	USA	1.15E+08
SRR27117437	Human	USA	1.15E+08
SRR27117438	Human	USA	8.64E+07
SRR27117439	Human	USA	1.02E+08
SRR27117440	Human	USA	9.37E+07
SRR27117442	Human	USA	1.21E+08
SRR27117443	Human	USA	1.11E+08
SRR27117444	Human	USA	1.06E+08
SRR27117445	Human	USA	1.08E+08
SRR27117446	Human	USA	1.02E+08
SRR27117447	Human	USA	1.11E+08
SRR27117448	Human	USA	1.08E+08
SRR27117449	Human	USA	1.13E+08
SRR27117450	Human	USA	1.03E+08
SRR27117451	Human	USA	1.12E+08
SRR27117452	Human	USA	1.19E+08
SRR27117453	Human	USA	1.17E+08
SRR27117454	Human	USA	1.04E+08
SRR27117455	Human	USA	1.20E+08
SRR27117456	Human	USA	6.02E+07
SRR27117457	Human	USA	1.01E+08
SRR27117458	Human	USA	9.82E+07
SRR27117459	Human	USA	1.07E+08

SRR27117460	Human	USA	1.09E+08
SRR27117461	Human	USA	1.13E+08
SRR27117462	Human	USA	6.41E+07
SRR27117463	Human	USA	9.26E+07
SRR28511868	Human	South Korea	2.53E+06
SRR28511879	Human	South Korea	6.75E+07
SRR28511909	Human	South Korea	6.52E+07
SRR28511931	Human	South Korea	6.72E+07
SRR28511952	Human	South Korea	7.00E+07
SRR28511963	Human	South Korea	6.25E+07
SRR28511974	Human	South Korea	6.51E+07
SRR28511985	Human	South Korea	6.80E+07
SRR28511996	Human	South Korea	6.61E+07
SRR28512006	Human	South Korea	6.58E+07
SRR28512017	Human	South Korea	6.82E+07
SRR28512028	Human	South Korea	6.77E+07
SRR28512039	Human	South Korea	3.28E+07
SRR28512040	Human	South Korea	6.79E+07
SRR28512041	Human	South Korea	7.03E+07
SRR10378023	Chicken	China	4.45E+07
SRR10378024	Chicken	China	4.75E+07
SRR10378025	Chicken	China	4.90E+07
SRR10378026	Chicken	China	4.67E+07
SRR10378027	Chicken	China	4.29E+07
SRR10378028	Chicken	China	4.70E+07
SRR10378029	Chicken	China	4.39E+07
SRR10378030	Chicken	China	4.62E+07
SRR10378031	Chicken	China	4.37E+07
SRR10378032	Chicken	China	4.40E+07
SRR10378033	Chicken	China	4.95E+07
SRR10378034	Chicken	China	4.43E+07
SRR10378035	Chicken	China	4.03E+07
SRR10378036	Chicken	China	4.89E+07
SRR10378037	Chicken	China	4.72E+07
SRR10378038	Chicken	China	5.27E+07
SRR10378039	Chicken	China	5.20E+07
SRR10378040	Chicken	China	4.77E+07
SRR10378041	Chicken	China	4.28E+07
SRR10378042	Chicken	China	4.30E+07
SRR10378043	Chicken	China	4.85E+07
SRR10378044	Chicken	China	4.62E+07
SRR10378045	Chicken	China	4.86E+07
SRR10378046	Chicken	China	4.05E+07
SRR10378047	Chicken	China	4.94E+07

SRR10378048	Chicken	China	3.51E+07
SRR10378049	Chicken	China	4.15E+07
SRR10378050	Chicken	China	4.82E+07
SRR10378051	Chicken	China	4.32E+07
SRR10378052	Chicken	China	5.85E+07
SRR10378053	Chicken	China	3.39E+07
SRR10378054	Chicken	China	4.73E+07
SRR10378055	Chicken	China	4.39E+07
SRR10378056	Chicken	China	4.11E+07
SRR10378057	Chicken	China	4.41E+07
SRR10378058	Chicken	China	4.01E+07
SRR10378059	Chicken	China	4.51E+07
SRR10378060	Chicken	China	4.96E+07
SRR10378061	Chicken	China	4.04E+07
SRR10378062	Chicken	China	5.97E+07
SRR10378063	Chicken	China	5.60E+07
SRR10378064	Chicken	China	5.73E+07
SRR10378065	Chicken	China	4.40E+07
SRR10378066	Chicken	China	4.71E+07
SRR10378067	Chicken	China	4.16E+07
SRR10378068	Chicken	China	4.51E+07
SRR10378069	Chicken	China	4.78E+07
SRR10378070	Chicken	China	3.83E+07
SRR10378071	Chicken	China	5.04E+07
SRR10378072	Chicken	China	4.99E+07
SRR10378073	Chicken	China	5.21E+07
SRR10378074	Chicken	China	5.87E+07
SRR10378075	Chicken	China	5.45E+07
SRR10378076	Chicken	China	5.79E+07
SRR10378077	Chicken	China	4.52E+07
SRR10378078	Chicken	China	4.65E+07
SRR10378079	Chicken	China	4.48E+07
SRR10378080	Chicken	China	4.69E+07
SRR10378081	Chicken	China	3.80E+07
SRR10378082	Chicken	China	4.57E+07
SRR14369131	Pig	Canada	1.36E+07
SRR14369132	Pig	Canada	8.36E+06
SRR14369133	Pig	Canada	7.48E+06
SRR14369134	Pig	Canada	2.71E+07
SRR14369135	Pig	Canada	2.40E+07
SRR14369136	Pig	Canada	3.07E+07
SRR14369137	Pig	Canada	1.39E+07
SRR14369138	Pig	Canada	1.71E+07
SRR14369139	Pig	Canada	1.80E+07

SRR14369140	Pig	Canada	1.92E+07
SRR14369142	Pig	Canada	7.25E+06
SRR14369143	Pig	Canada	8.91E+06
SRR14369144	Pig	Canada	2.62E+07
SRR14369145	Pig	Canada	5.97E+06
SRR14369146	Pig	Canada	2.80E+07
SRR14369147	Pig	Canada	1.91E+07
SRR14369148	Pig	Canada	2.71E+07
SRR14369149	Pig	Canada	4.75E+06
SRR14369150	Pig	Canada	1.70E+07
SRR14369151	Pig	Canada	1.89E+07
SRR14369152	Pig	Canada	1.27E+07
SRR14369153	Pig	Canada	1.25E+07
SRR14369154	Pig	Canada	1.66E+07
SRR14369155	Pig	Canada	3.10E+07
SRR14369156	Pig	Canada	2.70E+07
SRR14369157	Pig	Canada	1.98E+07
SRR14369158	Pig	Canada	1.47E+07
SRR14369159	Pig	Canada	9.66E+06
SRR14369160	Pig	Canada	2.97E+07
SRR14369161	Pig	Canada	1.90E+07
SRR14369162	Pig	Canada	2.06E+07
SRR14369163	Pig	Canada	1.59E+07
SRR14369164	Pig	Canada	1.31E+07
SRR14369165	Pig	Canada	1.74E+07
SRR14369166	Pig	Canada	1.21E+07
SRR14369167	Pig	Canada	2.29E+07
SRR14369168	Pig	Canada	2.44E+07
SRR14369169	Pig	Canada	2.36E+07
SRR14369170	Pig	Canada	2.59E+07
SRR14369171	Pig	Canada	9.73E+06
SRR14369172	Pig	Canada	1.44E+07
SRR14369173	Pig	Canada	1.02E+07
SRR14369174	Pig	Canada	1.08E+07
SRR14369175	Pig	Canada	8.16E+06
SRR14369176	Pig	Canada	1.61E+07
SRR14369177	Pig	Canada	4.38E+06
SRR14369178	Pig	Canada	3.34E+07
SRR14369179	Pig	Canada	1.71E+07
SRR14369180	Pig	Canada	1.95E+07
SRR14369181	Pig	Canada	9.75E+06
SRR14369182	Pig	Canada	2.62E+07
SRR14369183	Pig	Canada	2.29E+07
SRR14369184	Pig	Canada	1.74E+07

SRR14369185	Pig	Canada	3.52E+07
SRR14369186	Pig	Canada	1.27E+07
SRR14369187	Pig	Canada	3.79E+06
SRR14369188	Pig	Canada	2.71E+07
SRR14369190	Pig	Canada	7.35E+06
SRR14369191	Pig	Canada	6.55E+06
SRR14369192	Pig	Canada	2.17E+07
SRR14369193	Pig	Canada	1.78E+07
SRR14369194	Pig	Canada	2.84E+07
SRR14369195	Pig	Canada	1.98E+07
SRR14369197	Pig	Canada	1.94E+06
SRR14369198	Pig	Canada	2.52E+07
SRR14369199	Pig	Canada	2.52E+07
SRR14369200	Pig	Canada	1.59E+07
SRR14369201	Pig	Canada	3.14E+07
SRR14369202	Pig	Canada	3.34E+07
SRR14369203	Pig	Canada	3.52E+07
SRR14369204	Pig	Canada	1.64E+07
SRR14369205	Pig	Canada	3.28E+07
SRR14369206	Pig	Canada	3.82E+07
SRR14369207	Pig	Canada	1.35E+07
SRR14369208	Pig	Canada	3.12E+07
SRR14369209	Pig	Canada	2.00E+07
SRR14369210	Pig	Canada	2.39E+07
SRR14369211	Pig	Canada	2.31E+07
SRR14369212	Pig	Canada	8.51E+06
SRR14369213	Pig	Canada	4.90E+06
SRR14369214	Pig	Canada	1.74E+07
SRR14369215	Pig	Canada	2.83E+07
SRR14369216	Pig	Canada	1.97E+07
SRR14369217	Pig	Canada	1.52E+07
SRR14369218	Pig	Canada	3.47E+07
SRR14369219	Pig	Canada	2.23E+07
SRR14369220	Pig	Canada	2.00E+07
SRR14369221	Pig	Canada	2.92E+07
SRR14369223	Pig	Canada	3.11E+07
SRR14369224	Pig	Canada	7.43E+06
SRR14369225	Pig	Canada	3.39E+07
SRR14369226	Pig	Canada	2.97E+07
SRR14369227	Pig	Canada	2.31E+07
SRR14369228	Pig	Canada	1.51E+07
SRR14369229	Pig	Canada	1.02E+07
SRR14369232	Pig	Canada	1.45E+07
SRR14369233	Pig	Canada	1.38E+07

SRR14369234	Pig	Canada	2.18E+07
SRR14369235	Pig	Canada	1.09E+07
SRR14369236	Pig	Canada	4.07E+07
SRR14369238	Pig	Canada	5.62E+06
SRR14369240	Pig	Canada	1.23E+07
SRR14369241	Pig	Canada	3.85E+06
SRR14369242	Pig	Canada	2.33E+07
SRR14369243	Pig	Canada	3.11E+07
SRR14369244	Pig	Canada	2.30E+07
SRR14369245	Pig	Canada	2.49E+07
SRR14369246	Pig	Canada	1.95E+07
SRR14369247	Pig	Canada	8.42E+06
SRR14369248	Pig	Canada	1.00E+07
SRR14369249	Pig	Canada	8.73E+06
SRR14369250	Pig	Canada	1.12E+07
SRR14369251	Pig	Canada	5.76E+06
SRR14369253	Pig	Canada	1.72E+07
SRR14369254	Pig	Canada	8.62E+06
SRR14369255	Pig	Canada	2.79E+07
SRR14369256	Pig	Canada	1.50E+07
SRR14369257	Pig	Canada	3.35E+07
SRR14369258	Pig	Canada	2.65E+07
SRR14369259	Pig	Canada	2.69E+07
SRR14369260	Pig	Canada	1.77E+07
SRR14369261	Pig	Canada	1.59E+07
SRR14369262	Pig	Canada	6.21E+06
SRR14369263	Pig	Canada	9.66E+06
SRR14369264	Pig	Canada	1.26E+07
SRR14369265	Pig	Canada	1.10E+07
SRR14369266	Pig	Canada	3.14E+07
SRR14369267	Pig	Canada	2.17E+07
SRR14369268	Pig	Canada	2.19E+07
SRR14369269	Pig	Canada	2.35E+07
SRR14369270	Pig	Canada	8.42E+06
SRR14369271	Pig	Canada	2.96E+06
SRR14369272	Pig	Canada	2.23E+07
SRR14369273	Pig	Canada	3.38E+07
SRR14369274	Pig	Canada	2.27E+07
SRR14369276	Pig	Canada	7.14E+06
SRR14369277	Pig	Canada	1.86E+07
SRR14369278	Pig	Canada	1.32E+07
SRR14369279	Pig	Canada	2.51E+07
SRR14369280	Pig	Canada	3.83E+07
SRR14369281	Pig	Canada	1.86E+07

SRR14369282	Pig	Canada	5.67E+06
SRR14369283	Pig	Canada	2.24E+07
SRR14369284	Pig	Canada	5.80E+06
SRR14369285	Pig	Canada	6.01E+06
SRR14369286	Pig	Canada	1.90E+07
SRR14369287	Pig	Canada	3.33E+07
SRR14369288	Pig	Canada	6.97E+06
SRR14369289	Pig	Canada	2.49E+07
SRR14369291	Pig	Canada	1.09E+07
SRR14369292	Pig	Canada	9.85E+06
SRR14369293	Pig	Canada	1.02E+07
SRR14369294	Pig	Canada	2.81E+07
SRR14369295	Pig	Canada	1.48E+07
SRR14369296	Pig	Canada	2.92E+07
SRR14369297	Pig	Canada	1.91E+07
SRR14369298	Pig	Canada	9.62E+06
SRR14369299	Pig	Canada	3.16E+06
SRR14369300	Pig	Canada	4.25E+06
SRR14369301	Pig	Canada	1.63E+07
SRR14369302	Pig	Canada	1.24E+07
SRR14369303	Pig	Canada	1.32E+07
SRR14369304	Pig	Canada	7.67E+06
SRR14369305	Pig	Canada	2.74E+07
SRR14369306	Pig	Canada	2.40E+07
SRR14369307	Pig	Canada	2.46E+07
SRR14369308	Pig	Canada	2.00E+07
SRR14812367	Pig	Canada	3.46E+07
SRR14812368	Pig	Canada	3.46E+07
SRR14812369	Pig	Canada	3.47E+07
SRR14812370	Pig	Canada	3.45E+07
SRR14812371	Pig	Canada	3.44E+07
SRR14812372	Pig	Canada	3.45E+07
SRR14812373	Pig	Canada	3.32E+07
SRR14812374	Pig	Canada	3.36E+07
SRR14812375	Pig	Canada	3.66E+07
SRR14812376	Pig	Canada	3.37E+07
SRR14812377	Pig	Canada	3.56E+07
SRR14812378	Pig	Canada	3.76E+07
SRR17317403	Pig	China	5.42E+07
SRR17317404	Pig	China	4.70E+07
SRR17317405	Pig	China	5.37E+07
SRR17317406	Pig	China	4.50E+07
SRR17317407	Pig	China	4.87E+07
SRR17317408	Pig	China	3.70E+07

SRR17317409	Pig	China	5.09E+07
SRR17317410	Pig	China	4.52E+07
SRR17317411	Pig	China	3.73E+07
SRR17317412	Pig	China	4.44E+07
SRR17317413	Pig	China	3.53E+07
SRR17317414	Pig	China	4.64E+07
SRR17317415	Pig	China	4.88E+07
SRR17317416	Pig	China	3.81E+07
SRR17317418	Pig	China	5.05E+07
SRR17317419	Pig	China	3.37E+07
SRR17317420	Pig	China	3.88E+07
SRR17317421	Pig	China	4.61E+07
SRR17317422	Pig	China	4.54E+07
SRR17317423	Pig	China	1.67E+07
SRR17317424	Pig	China	4.63E+07
SRR17317425	Pig	China	4.33E+07
SRR17317426	Pig	China	3.89E+07
SRR4096731	Chicken	China	4.99E+07
SRR4096732	Chicken	China	6.11E+07
SRR4096733	Chicken	China	3.83E+07
SRR4096734	Chicken	China	4.22E+07
SRR4096735	Chicken	China	1.04E+07
SRR4096736	Chicken	China	5.39E+07
SRR4096737	Chicken	China	5.12E+07
SRR4096738	Chicken	China	4.94E+07
SRR4096739	Chicken	China	5.62E+07
SRR4096740	Chicken	China	6.25E+07
SRR4096741	Chicken	China	5.79E+07
SRR4096742	Chicken	China	4.48E+07
SRR4096743	Chicken	China	5.66E+07
SRR4096744	Chicken	China	4.41E+07
SRR4096745	Chicken	China	5.95E+07
SRR4096746	Chicken	China	4.81E+07
SRR4096747	Chicken	China	4.96E+07
SRR4096748	Chicken	China	6.88E+07
SRR4096749	Chicken	China	4.45E+07
SRR4096750	Chicken	China	6.00E+07
SRR4096751	Chicken	China	7.52E+06
SRR4096752	Chicken	China	6.21E+07
SRR4096753	Chicken	China	5.14E+07
SRR4096754	Chicken	China	4.27E+07
SRR4096755	Chicken	China	1.85E+07
SRR4096756	Chicken	China	5.09E+07
SRR4096757	Chicken	China	4.75E+07

SRR4096758	Chicken	China	4.77E+07
SRR4096759	Chicken	China	9.33E+06
SRR9113688	Chicken	UK	2.94E+07
SRR9113689	Chicken	UK	2.36E+07
SRR9113690	Chicken	UK	1.53E+07
SRR9113691	Chicken	UK	4.42E+07
SRR9113692	Chicken	UK	2.44E+07
SRR9113693	Chicken	UK	3.16E+07
SRR9113694	Chicken	UK	5.58E+06
SRR9113695	Chicken	UK	3.87E+06
SRR9113696	Chicken	UK	3.88E+07
SRR9113697	Chicken	UK	2.74E+07
SRR9113698	Chicken	UK	6.24E+07
SRR9113699	Chicken	UK	4.76E+07
SRR9113700	Chicken	UK	2.35E+07
SRR9113701	Chicken	UK	1.98E+07
SRR9113702	Chicken	UK	4.52E+07
SRR9113703	Chicken	UK	8.96E+06
SRR9113704	Chicken	UK	4.87E+07
SRR9113705	Chicken	UK	5.53E+07
SRR9113706	Chicken	UK	3.79E+07
SRR9113707	Chicken	UK	3.54E+07
SRR9113708	Chicken	UK	5.40E+07
SRR9113709	Chicken	UK	2.61E+07
SRR9113710	Chicken	UK	7.40E+07
SRR9113712	Chicken	UK	7.47E+07
SRR9113713	Chicken	UK	4.57E+07
SRR9113714	Chicken	UK	3.53E+07
SRR9113715	Chicken	UK	1.47E+07
SRR9113716	Chicken	UK	6.22E+07
SRR9113717	Chicken	UK	2.11E+07
SRR9113718	Chicken	UK	5.04E+06
SRR9113719	Chicken	UK	1.86E+07
SRR9113720	Chicken	UK	2.75E+07
SRR9113721	Chicken	UK	2.55E+07
SRR9113722	Chicken	UK	6.37E+07
SRR9113723	Chicken	UK	5.28E+07
SRR9113724	Chicken	UK	6.37E+07
SRR9113725	Chicken	UK	3.67E+07
SRR9113726	Chicken	UK	5.42E+07
SRR9113727	Chicken	UK	9.82E+06
SRR9113728	Chicken	UK	6.43E+07
SRR9113729	Chicken	UK	4.94E+07
SRR9113730	Chicken	UK	2.03E+07

SRR9113731	Chicken	UK	6.34E+07
SRR9113733	Chicken	UK	2.31E+07
SRR9113735	Chicken	UK	7.74E+07
SRR9113736	Chicken	UK	3.21E+07
SRR9113737	Chicken	UK	3.65E+07
Total			5.90E+10
SD			3.64E+07
Average			4.63E+07

Supplementary Table 2: Disease or condition associated with shared SGBs

**Species-level genome bins (SGBs)**	**Disease or condition caused in humans or animals**
Alistipes ihumii	Anorexia (Parker et al. 2020).
Alistipes putredinis	Acute appendicitis, abdominal and rectal abscesses, foot rot in sheep (Parker et al., 2020).
Anaerotruncus colihominis	Generally commensal; rare cause of bacteraemia in immunocompromised patients (Lau et al. 2006).
Bacteroides caccae	Gut commensal; opportunistic pathogen in intra-abdominal abscesses and bacteraemia (Wexler 2007).
Bacteroides fragilis	Major opportunistic pathogen - leading anaerobe in intra-abdominal infections, peritonitis, bacteraemia, and abscesses. Enterotoxigenic strains (ETBF) cause diarrhoea and are linked to colorectal cancer (Sears 2009; Wexler 2007).
Bacteroides thetaiotaomicron	Predominantly a beneficial gut commensal; opportunistic pathogen in intra-abdominal abscesses and bacteraemia (Wexler 2007).
Citrobacter freundii	Opportunistic pathogen - causes urinary tract infections, neonatal illness, bacteraemia, intra-abdominal infections (Jabeen et al. 2023).
Clostridium disporicum	Gut commensal with opportunistic intra-abdominal infections (Plassart et al. 2013).
Clostridium perfringens	Major pathogen - causes gas gangrene (clostridial myonecrosis), food poisoning, necrotic enteritis, enterotoxaemia in livestock (notably broiler chickens), and antibiotic-associated diarrhoea (Immerseel et al. 2004).
Eggerthella lenta	Opportunistic pathogen - causes bacteraemia (notably polymicrobial), and intra-abdominal infections (Gardiner et al. 2015).
Enterobacter hormaechei SGB10130 group	Significant nosocomial pathogen - causes bloodstream infections, ventilator-associated pneumonia, urinary tract infections, neonatal sepsis; frequent carbapenem-resistant strains (Annavajhala et al. 2019).
Enterococcus cecorum	Major emerging poultry pathogen - causes vertebral osteoarthritis/spondylitis ("kinky back"), femoral head necrosis, sepsis, and lameness in broiler chickens; rare opportunistic infections in humans (Jung et al. 2018).
Enterococcus faecalis	Major opportunistic pathogen - causes urinary tract infections, endocarditis, bacteraemia, surgical wound infections, and neonatal sepsis. Associated with antibiotic resistance (vancomycin-resistant enterococci, VRE) (Arias and Murray, 2012, Nat Rev Microbiol).
Enterococcus faecium	Major nosocomial pathogen - causes endocarditis, bacteraemia, urinary tract infections; leading cause of VRE infections globally (Arias and Murray 2012).
Enterococcus gallinarum	Opportunistic human pathogen, nosocomial infections, bacteremia, urinary tract and surgical wound infections (Monticelli et al. 2018).
Escherichia coli	Major pathogen - causes urinary tract infections, neonatal meningitis, sepsis, gastroenteritis. In poultry, avian pathogenic *E. coli* (APEC) causes colibacillosis, airsacculitis, and yolk sac infections (Christensen et al. 2021; Kaper et al. 2004).
Klebsiella pneumoniae	Major pathogen - causes pneumonia (community-acquired and nosocomial), urinary tract infections, bacteraemia, liver abscesses, and neonatal sepsis (Paczosa and Mecsas 2016).
Proteus mirabilis	Major uropathogen - causes complicated urinary tract infections, catheter-associated infections, and bacteraemia (Chen et al. 2012).

References

Annavajhala MK, Gomez-Simmonds A, Uhlemann A-C (2019) Multidrug-resistant *Enterobacter cloacae* complex emerging as a global, diversifying threat. Front Microbiol 10:44

Arias CA, Murray BE (2012) The rise of the *Enterococcus*: beyond vancomycin resistance. Nat Rev Micro 10(4):266-278

Chen C-Y, Chen Y-H, Lu P-L, Lin W-R, Chen T-C, Lin C-Y (2012) *Proteus mirabilis* urinary tract infection and bacteremia: risk factors, clinical presentation, and outcomes. J Microbiol Immunol Infect 45(3):228-236

Christensen H, Bachmeier J, Bisgaard M (2021) New strategies to prevent and control avian pathogenic *Escherichia coli* (APEC). Avian Pathol 50(5):370-381

Gardiner BJ, Tai A, Kotsanas D, Francis MJ, Roberts SA, Ballard SA, Junckerstorff RK, Korman TM (2015) Clinical and microbiological characteristics of *Eggerthella lenta* bacteremia. J Clin Micro 53(2):626-635

Immerseel FV, Buck JD, Pasmans F, Huyghebaert G, Haesebrouck F, Ducatelle R (2004) *Clostridium perfringens* in poultry: an emerging threat for animal and public health. Avian Pathol 33(6):537-549

Jabeen I, Islam S, Hassan AI, Tasnim Z, Shuvo SR (2023) A brief insight into *Citrobacter* species-a growing threat to public health. Front. Antibiot 2:1276982

Jung A, Chen LR, Suyemoto MM, Barnes HJ, Borst LB (2018) A review of *Enterococcus cecorum* infection in poultry. Avian Dis 62(3):261-271

Kaper JB, Nataro JP, Mobley HL (2004) Pathogenic *Escherichia coli*. Nat Rev Micro 2(2):123-140

Lau SK, Woo PC, Woo GK, Fung AM, Ngan AH, Song Y, Liu C, Summanen P, Finegold SM, Yuen K-y (2006) Bacteraemia caused by *Anaerotruncus colihominis* and emended description of the species. J Clin Pathol 59(7):748-752

Monticelli J, Knezevich A, Luzzati R, Di Bella S (2018) Clinical management of non-faecium non-faecalis vancomycin-resistant enterococci infection. Focus on *Enterococcus gallinarum* and *Enterococcus casseliflavus/flavescens*. J Infect Chemother 24(4):237-246

Paczosa MK, Mecsas J (2016) *Klebsiella pneumoniae*: going on the offense with a strong defense. Microbiol Mol Biol Rev 80(3):629-661

Parker BJ, Wearsch PA, Veloo AC, Rodriguez-Palacios A (2020) The genus *Alistipes*: gut bacteria with emerging implications to inflammation, cancer, and mental health. Front Immunol 11:906

Plassart C, Mauvais F, Heurté J, Sautereau J, Legeay C, Bouvet P (2013) First case of intra-abdominal infection with *Clostridium disporicum*. Anaerobe 19:77-78

Sears CL (2009) Enterotoxigenic *Bacteroides fragilis*: a rogue among symbiotes. Clin Microbiol Rev 22(2):349-369

Wexler HM (2007) *Bacteroides*: the good, the bad, and the nitty-gritty. Clin Microbiol Rev 20(4):593-621
